# Ginsenoside Profiles of Wild-Simulated and Forest-Grown *Panax ginseng*: A Scoping Review of Quantitative Evidence and Confounding Factors

**DOI:** 10.3390/plants15213472

**Published:** 2026-11-06

**Authors:** Min-Kyu Park, Jong-Du Lee, Gun-Myung Lee, Yeong-Jun Park, Xiaofei Dang, Yuxin He, Hyuk-Joon Kwon, Jae-Ho Shin

**Affiliations:** 1 Daesang Holdings, Jecheon-si 27116, Republic of Korea; pmk601313@daesang.com (M.-K.P.); jdlee1015@daesang.com (J.-D.L.); leegm@daesang.com (G.-M.L.); 2 Department of Applied Biosciences, Kyungpook National University, Daegu 41566, Republic of Korea; yjpark1091@knu.ac.kr; 3 KNU NGS Core Facility, Kyungpook National University, Daegu 41566, Republic of Korea; 4 Land Free (Suzhou) Biotechnology Co., Ltd., Suzhou 215000, China; dangxiaofei@landfreebio.com (X.D.); heyuxin@landfreebio.com (Y.H.)

**Keywords:** *Panax ginseng*, ginsenosides, wild-simulated ginseng, confounding factors, evidence synthesis

## Abstract

Wild-simulated and forest-grown *Panax ginseng* commands substantially higher prices than field-cultivated ginseng, largely on the assumption of a superior ginsenoside profile. Following PRISMA-ScR, we searched PubMed and Crossref (14 August 2026) and hand-searched Korean-language journals; 270 of 300 records were retained, and 4199 ginsenoside values were extracted from 44 studies, with the study as the unit of synthesis. Of 18 within-study contrasts in *P. ginseng*, eight reported higher major-ginsenoside abundances in forest material, but 13 of the 18 contrasts were not age-matched. The five age-matched comparisons were mixed: higher relative abundance of 19 constituents in one untargeted study, higher Re only in another, higher totals but lower Rg1 and Rf in a third, and rare-ginsenoside differences in two, with major ginsenosides either not consistently different or not reported. Truly wild ginseng is represented by fewer than ten quantified roots. Three conclusions follow: (i) forest-grown roots are chemically distinguishable, but consistent enrichment of major ginsenosides has not been shown once age is considered; (ii) largely uncontrolled age, site, plant part, and germplasm produce variation comparable to the reported system differences; and (iii) age-matched, germplasm-controlled studies with harmonized reporting are needed before profile-based quality claims are justified.

## 1. Introduction

Ginseng root is among the world’s most valuable specialty crops, and its market value is stratified by cultivation system. The premium grades are forest-based: in Korea, sanyangsam—seed or seedlings placed in standing forest and left with minimal management for seven to twenty years—and in China, the equivalent lín xià shēn. These are contrasted with field-cultivated (garden) ginseng, harvested at four to six years under artificial shade. Above them sits genuinely wild ginseng, sansam, which is foraged rather than cultivated. The premium attached to forest-based grades rests on the belief that they yield chemically superior roots. Because the pharmacological identity of ginseng is conventionally attributed to its triterpenoid saponins, the ginsenosides—which show a broad spectrum of reported bioactivities, including anti-inflammatory, antioxidant, neuroprotective, cardiovascular, immunomodulatory, antidiabetic, and anticancer effects [1,2]—this belief is in principle testable against the published quantitative record. Whether the record supports it—and under what caveats—is the central question this review addresses. Field-cultivated ginseng appears throughout as the reference against which forest-based grades are measured, not as a co-equal subject; the corpus was assembled around the wild and forest-ginseng axis and does not claim to review the field-ginseng literature in its own right (Section 2.1).

Ginsenosides are not unique to *P. ginseng*. At least 289 saponins had been reported from eleven *Panax* species by 2012, including *P. quinquefolius*, *P. notoginseng*, and *P. japonicus* [3], and dammarane-type saponins closely related to the ginsenosides also occur outside the Araliaceae, most prominently as the gypenosides of *Gynostemma* [4]. We found no comparative quantitative evidence that other Araliaceae are richer sources. *P. ginseng* remains the reference species from a pharmaceutical perspective because it combines a diverse complement of protopanaxadiol- and protopanaxatriol-type ginsenosides [5] with a long history of medicinal use and the most extensive basic and clinical research [1], and because its cultivation-system grades carry the price differences examined here. Other *Panax* species are therefore used below only as context.

Three developments make a scoping synthesis timely. First, the primary literature has expanded rapidly in recent years: in the corpus assembled here (Section 2), 54% of records were published in 2021 or later, following the most recent mapping of the wild-simulated ginseng literature—a bibliometric analysis to 2020 that quantified publication patterns but did not synthesize chemical measurements [6]. Second, analytical practice has diversified—from high-performance liquid chromatography with ultraviolet detection (HPLC-UV) using a handful of reference standards to liquid chromatography–tandem mass spectrometry (LC–MS/MS) panels quantifying 15–21 ginsenosides, two-dimensional liquid chromatography (2D-LC) capable of measuring over 200 ginsenosides [7], and high-resolution untargeted profiling of several hundred saponins [8]. Paradoxically, this diversification makes naïve cross-study comparison less, not more, reliable. Third, primary studies have begun to analyze the root-associated microbiome and soil environment jointly with ginsenoside content [9,10,11], but these associations have not yet been charted across the literature.

The most directly relevant recent dataset—an age series of garden and wild-simulated ginseng profiled for 359 ginsenosides in one region [12]—is examined in Section 5.2. It is noted here because its authors framed the problem in the terms used in this review: most comparisons have set older forest roots against younger field roots, so that cultivation system and age are confounded.

Parts of this ground have been reviewed before. A Chinese-language narrative review compared forest-cultivated and garden ginseng across morphology, chemical composition, and pharmacology [13], and the quality-control and authentication literature has been surveyed repeatedly. None of these, however, tabulated the reported content values or examined whether they can be compared across studies. This review therefore differs from existing syntheses in three respects: it treats cultivation system as the agronomic unit of comparison; it charts the quantitative record with explicit attention to whether reported values are comparable, including confounding by age and germplasm; and it incorporates Korean- and Chinese-language primary literature retrieved through Crossref and hand-searching, which reports a substantial share of the world’s wild-simulated and forest-cultivated ginseng measurements but is rarely cited in English-language reviews. Because these sources were not searched systematically—CNKI, Wanfang, and VIP were not queried, and Korean-language records were identified by hand-searching—this review describes the record retrievable through the search arms of Section 2.1 rather than the complete multilingual literature (Section 2.3).

We ask four questions: (i) What do within-study comparisons report about profile differences among cultivation systems, and in which direction? (ii) How do cultivation age, plant part, site, and germplasm modify these differences? (iii) Which methodological factors limit comparability, and how should future studies report? (iv) What environmental and microbiome factors are associated with profile variation, and what would it take to translate association into forest-farming practice? Processing-induced transformations and in vitro production systems are covered only as boundary conditions (Section 9), as they are reviewed elsewhere [14,15].

## 2. Scope, Search, and Charting

### 2.1. Search and Screening

This review followed the PRISMA extension for scoping reviews (PRISMA-ScR) [16]; the completed checklist is provided as Supplementary Table S3. The review protocol was not registered in advance (for example, on the Open Science Framework or with JBI). The subject of the search is forest-based ginseng: every arm is anchored on the wild and forest-ginseng axis, with field-cultivated ginseng entering only through within-study contrasts reported in those records. We therefore make no claim to have reviewed the field-ginseng literature in its own right; the comparisons below should be read as “what the forest-ginseng literature reports about itself relative to field ginseng”. The literature base was assembled in four arms, all run on 14 August 2026: (i) a PubMed query combining 14 wild/forest-ginseng terms (wild ginseng, wild-simulated, mountain-cultivated, forest-grown, mountainous-forest-cultivated, and related terms; title/abstract field) with ginsenoside or saponin terms (91 records); (ii) the same terms intersected with microbiome terms without requiring ginsenoside keywords (23 records); (iii) a Crossref bibliographic search for each wild/forest-ginseng phrase (100 records per query), retaining records whose titles contained the phrase, to recover journals not reached by the PubMed queries (220 records); and (iv) hand-searched records, including Korean-language journals indexed in the Korea Citation Index (18 records). No date or language limits were applied, and the complete search strings are given in Supplementary Table S2. After de-duplication (by normalized DOI, then normalized title), 300 unique records were identified.

**Figure 1:**
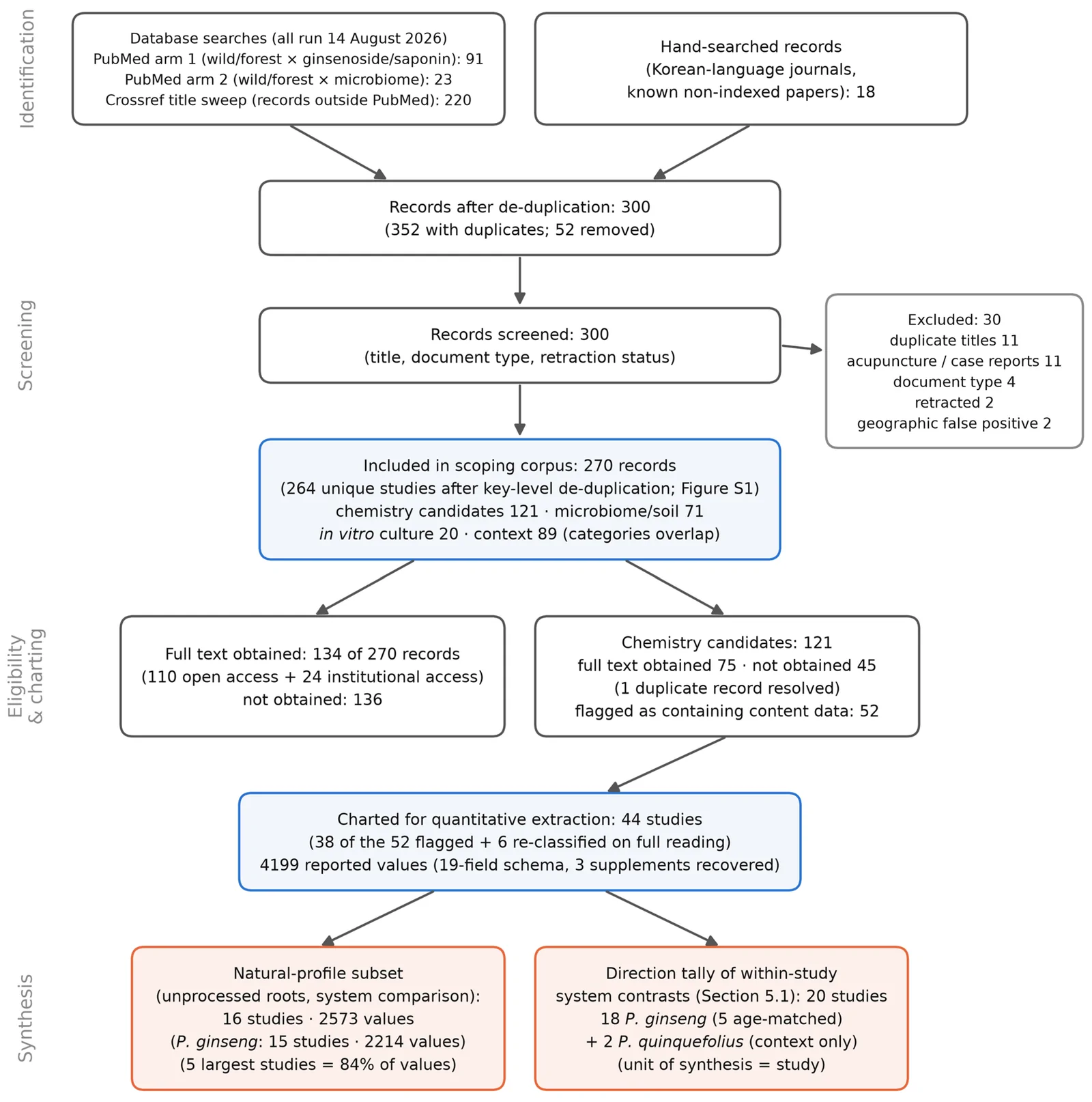
Flow of records through identification, screening, full-text retrieval, quantitative charting, and synthesis (PRISMA-ScR). Categories within the scoping corpus overlap (for example, a study may qualify both as a chemistry candidate and as a microbiome/soil study). Full texts were obtained through open-access routes (110) and institutional access (24); 45 chemistry candidates without accessible full texts could not be assessed. The 52 studies flagged by full-text scanning and the 44 studies charted differ because full reading removed 14 flagged studies and added 6 (Section 2.2). The direction tally (Section 5.1) also includes contrasts reported only as relative comparisons or verified at the abstract level. The unit of synthesis in Section 5, Section 6, Section 7 and Section 8 is the study, not the extracted value.

Records were eligible if their title or abstract concerned wild, wild-simulated, mountain-cultivated, or forest-grown ginseng (*P. ginseng*, or *P. quinquefolius* as context) and reported on ginsenoside or saponin chemistry, the rhizosphere or endophytic microbiome, soil, cultivation, authentication, or processing. Five predefined exclusion criteria were applied: non-article document types such as corrections, errata, and data sets (E1); retracted or withdrawn items (E2); geographic false positives in which “Changbai Mountain ginseng” denotes field-cultivated ginseng from that region (E3); clinical pharmacopuncture, acupuncture, and case reports (E4); and duplicate titles registered under different DOIs (E5). The exclusion rules were applied programmatically to titles and database metadata, and every inclusion and exclusion decision was then checked manually by one reviewer (M.-K.P.); title screening was therefore not performed in duplicate (Section 2.3). Following screening, 270 records were retained, and 30 were excluded (E1, 4; E2, 2; E3, 2; E4, 11; E5, 11); one retracted item carried its retraction only as a title prefix rather than in database metadata. Of the retained corpus, 62% of records (168 of 270) were retrieved only through Crossref or hand-searching rather than by the PubMed queries, and the only previous review of the area [6] was among them. This figure describes the reach of our PubMed queries, not MEDLINE indexing status: some of these records may be indexed in MEDLINE but were not matched by the query terms. At the charting stage, key-level de-duplication merged six further pairs of records that shared a DOI or a near-identical title, leaving 264 unique studies; Figure S1 describes these 264 unique studies, whereas the corpus-level counts elsewhere refer to the 270 retained records. Korean-language records were identified by hand-searching only, and we make no claim of systematic coverage of the Korean literature. The flow of records is shown in Figure 1.

### 2.2. Charting and Unit of Analysis

Full texts were obtained for 134 of the 270 retained records: 110 through open-access routes (PubMed Central/Europe PMC, open-access copies located via OpenAlex, and publisher websites) and 24 subscription-only papers through institutional library access; the figure of 134 therefore already includes the 24 institutionally accessed papers. Of the 121 chemistry candidates, 75 were retrieved in full, 45 could not be obtained, and one record was resolved as a duplicate during retrieval. Full-text eligibility for quantitative charting was assessed in two steps. First, each retrieved full text was scanned for unit-bearing ginsenoside contents, which flagged 52 of the 75 studies as likely to contain content data (23 with content tables confirmed in structured full-text XML and 29 with values detected in the text or PDF). Second, every study was read in full during extraction: 14 of the 52 flagged studies proved to contain no transcribable sample-level values (values shown only in figures, qualitative marker tables, pharmacokinetic data, or secondary compilations), whereas 6 studies that the scan had not flagged were found to contain usable content tables. This yielded the 44 studies charted for quantitative extraction (Figure 1).

From 44 studies, every reported ginsenoside value (4199 values, including supplementary tables from three studies) was transcribed by one author (M.-K.P.) and re-checked against the source by a second author (J.-D.L. or G.-M.L.). The unit of synthesis throughout is the study (or the within-study contrast), not the individual value: transcribed values are not independent observations. The five largest studies contribute 84% of the values in the natural-profile subset; one reports supplementary tables without printed units, and three originate from a single research group working at one site. We therefore report direction tallies and study-level anchors rather than pooled means, and we do not perform meta-analysis.

Values from processed material (steamed, fermented, enzymatically converted), in vitro cultures, soil and animal matrices, extraction-optimization experiments, and secondary products were charted but excluded from statements about natural profiles, as were three Ra1 values that duplicate other entries of the same source table (Appendix A, item 3). After this filter, 2573 values from 16 studies remained available for comparison between cultivation systems: 2214 values from 15 studies of *P. ginseng* and 359 values from *P. quinquefolius*. The other 28 charted studies contributed only such excluded material or presence-only records without printed contents. The direction tally in Section 5.1 is broader than this subset: it also includes within-study contrasts reported only as relative or untargeted comparisons, or verified at abstract level, which contribute a direction but no transcribed values.

Full-text verification also revealed that title- and abstract-level screening overestimates the quantitative evidence: of the 75 chemistry candidates retrieved in full, 31 (41%) yielded no transcribable values, reporting only relative (peak-area or ion-intensity) comparisons, graphical data, qualitative identifications, or no primary measurement. During transcription, internal inconsistencies were detected in several published tables (component sums not matching printed totals, duplicated entries, group sizes irreconcilable with individual-sample data). These are itemized in Appendix A (Table A1) and motivate the reporting recommendations in Section 12.

### 2.3. Limitations of the Search and Charting

The evidence base has explicit boundaries that constrain how far the findings can be generalized. First, Chinese-language literature was captured only through the Crossref title search and English-language metadata; CNKI, Wanfang, and VIP were not searched, so Chinese-language studies without matching Crossref-registered titles are missing. Second, Korean-language records were identified by hand-searching, and full coverage of the Korean literature cannot be guaranteed. Third, 136 of the 270 retained records, including 45 of the 121 chemistry candidates, could not be obtained in full, so the quantitative synthesis rests on the accessible subset. Fourth, the protocol was not registered in advance, and title screening relied on predefined rules verified by a single reviewer, although every transcribed value was re-checked by a second author. Fifth, the proportion of records retrieved outside PubMed (62%) reflects the reach of our queries rather than MEDLINE indexing. The conclusions of this review therefore describe the record retrievable through the four search arms and should not be generalized to the complete Chinese- and Korean-language literature.

## 3. Cultivation Systems and Terminology

Establishing terminology is a prerequisite for this review, because the same production system appears under different English names, and different systems are sometimes described by the same name (Table 1). Korean usage distinguishes natural wild ginseng (sansam), wild-simulated ginseng (sanyangsam; seed or seedlings placed in forest with essentially no further management), and field cultivation under artificial shade. Chinese literature distinguishes forest ginseng (lín xià shēn; “ginseng under the forest” or “mountainous forest-cultivated ginseng”) from garden ginseng, and market-purchased “wild ginseng” of uncertain provenance is a documented complication. One study titled on wild ginseng describes four-year-old market material that the authors elsewhere call cultivated [17], and comparisons of American ginseng frequently rely on root-scar counts to age putatively wild roots [18]. Because system labels carry price premia, mislabeling is both an economic and a scientific problem (Section 10).

The boundary between wild-simulated and forest-cultivated systems is porous—Korean sanyangsam and Chinese lín xià shēn are both frequently rendered “forest-grown”—and several studies pool them. Where a paper’s system label was ambiguous, we retained the author’s terminology and recorded the ambiguity rather than reclassifying the system label. Throughout this review, “forest-based systems” denotes the pooled category comprising wild-simulated and forest-cultivated ginseng.

One boundary deserves emphasis because it bears on the highest-value grade. Genuinely wild ginseng is foraged, not cultivated, and its quantitative record is very small. Two roots of estimated ages 50 and 100 years were quantified individually [8], and a Korean three-way comparison analyzed seven roots of estimated age 20–50 years, collected on Changbai Mountain and authenticated as wild by expert appraisal, reporting means of five after the highest and lowest values had been excluded [19]. Only the two individually reported roots were transcribed into our dataset (16 of the 2214 *P. ginseng* values in the natural-profile subset); the Korean study was charted for the direction tally at abstract level because its full text could not be obtained during charting, and it was read in full during revision. The grade commanding the largest premium therefore has a minimal quantitative chemical record—fewer than ten roots in two studies—which is itself a finding: statements about the composition of sansam in the trade and in the secondary literature are not currently traceable to published measurement. Accordingly, comparative conclusions regarding cultivation-system differences in *Panax ginseng* are restricted to wild-simulated and forest-cultivated material; evidence from wild American ginseng (*Panax quinquefolius*), the sister species, is included only for contextual or mechanistic evidence, not for inferring system superiority in *P. ginseng*.

## 4. Ginsenoside Classes in Brief

Ginsenosides are classified principally into protopanaxadiol (PPD; Rb1, Rb2, Rc, Rd, and derivatives), protopanaxatriol (PPT; Rg1, Re, Rf, and derivatives), and oleanane (Ro) groups. “Minor” ginsenosides (Rg3, Rg5, Rk1, Rh2, and compound K) occur at trace levels in raw roots and are enriched mainly through processing or microbial conversion [14]. Comparisons in this review therefore focus on the major ginsenosides quantifiable in raw material, their sums, and class ratios (PPD/PPT), which several discrimination studies identify as system-informative [20,21].

Transcriptomic comparisons of wild and cultivated plants across growth years suggest that growth year exerts comparatively modest effects on biosynthetic gene expression in wild ginseng [22]. In wild-simulated plants, comparison of 7- and 13-year roots indicates that pentose-phosphate and abscisic-acid-responsive genes, rather than core pathway genes, are age-responsive [23]. These findings indicate that between-system differences cannot be reduced to age alone.

## 5. The Quantitative Record

### 5.1. Direction of Within-Study Contrasts

**Figure 2:**
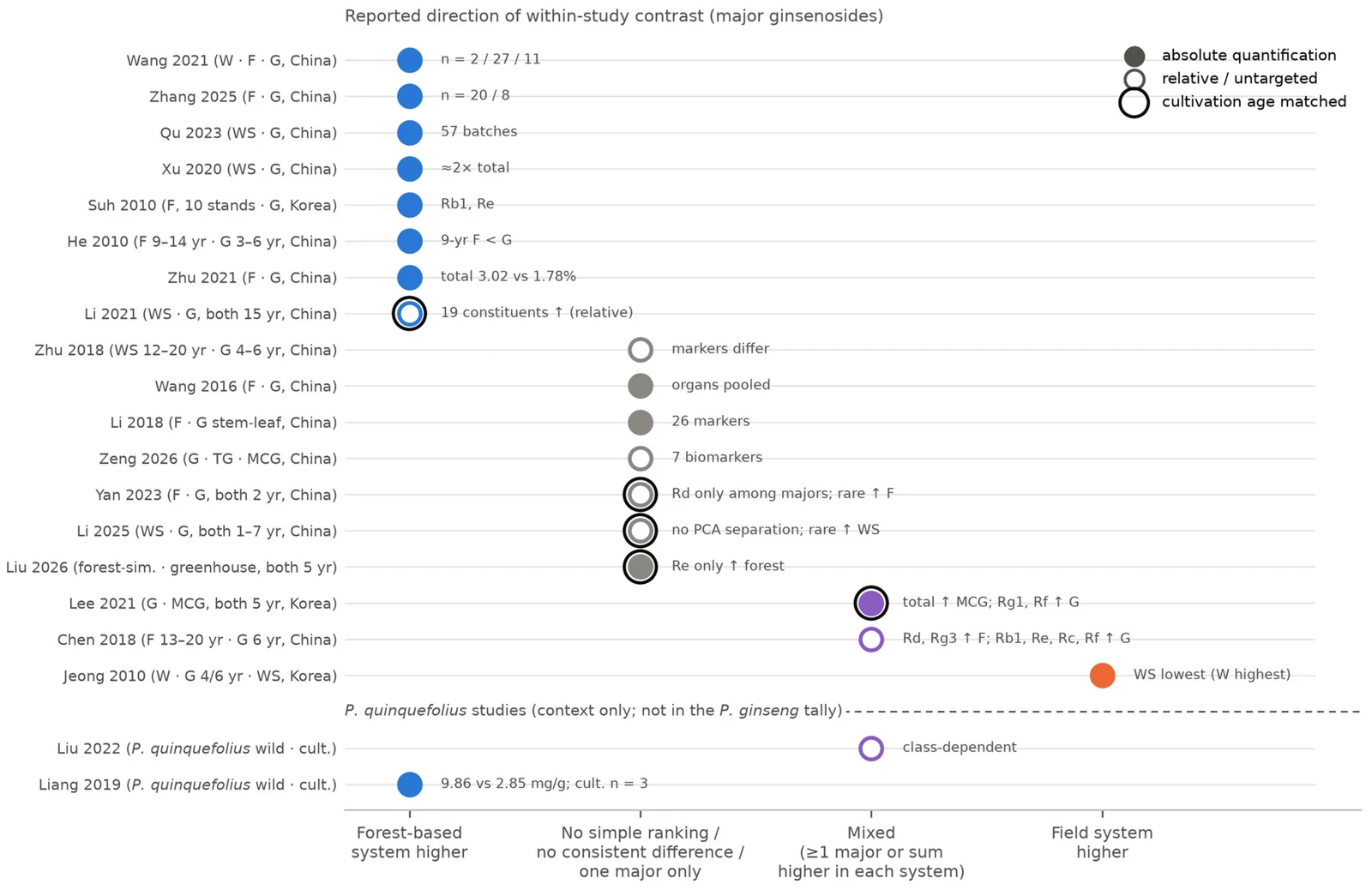
Reported direction of within-study contrasts between cultivation systems (one row per study; see Table 2), with the 18 *Panax ginseng* studies shown separately from the two *P. quinquefolius* studies, which are included as context only. Classes (defined in Section 5.1): forest-based system higher; mixed (at least one major ginsenoside, or their sum, higher in each system); field system higher; and no simple ranking (profiles distinguishable without a directional ranking of the major ginsenosides, no consistent difference in them, or a difference in only one major ginsenoside). For studies with both age-matched and age-unmatched arms, the age-matched arm determined the class. Filled markers indicate absolute quantification; open markers indicate relative or untargeted comparison; black rings mark the five comparisons in which cultivation age was matched. Abbreviations: W, wild; WS, wild-simulated; F, forest-cultivated; G, field (garden)-cultivated; TG, transplanted ginseng. Of the five age-matched comparisons, neither of the two with absolute quantification of the major ginsenosides showed a uniform enrichment in forest-based material, and the one forest-higher class among them rests on relative abundances in an untargeted comparison (Section 5.1). Studies, from top to bottom: Wang 2021 [8]; Zhang 2025 [20]; Qu 2023 [7]; Xu 2020 [30]; Suh 2010 [31]; He 2010 [29]; Zhu 2021 [36]; Li 2021 [28]; Zhu 2018 [32]; Wang 2016 [33]; Li 2018 [34]; Zeng 2026 [35]; Yan 2023 [25]; Li 2025 [12]; Liu 2026 [27]; Lee 2021 [26]; Chen 2018 [24]; Jeong 2010 [19]; Liu 2022 [18]; Liang 2019 [37].

Table 2 and Figure 2 summarize every within-study contrast between cultivation systems that we could verify at full-text or abstract level. Each contrast was assigned to one of four classes, with Rb1, Rb2, Rc, Rd, Re, Rf, and Rg1 taken as the major ginsenosides and differences counted as each study reported them under its own statistical or differential criteria (numerical trends alone did not count): forest higher, when two or more major ginsenosides or their sum were reported higher in forest-based material and none was reported higher in field material; field higher, when the reverse held; mixed, when at least one major ginsenoside, or their sum, was reported higher in each system; and no simple ranking, when the systems were described as distinguishable without a directional ranking of the major ginsenosides, showed no consistent difference in them, or differed in only one of them. For untargeted comparisons, the reported differential constituents were used; where several age classes were compared without an age-matched arm, the direction in most age classes was used; and wild roots were not used to assign the class, which reflects the forest-based arm against the field arm. The class records the direction of the differences reported by the authors, not their uniformity across ginsenosides. For studies with both age-matched and age-unmatched arms, the age-matched arm determined the class. Of the eighteen *P. ginseng* contrasts, eight were classed as forest higher, seven as no simple ranking, two as mixed, and one as field higher. The two *P. quinquefolius* studies are listed separately as context and are not included in these counts.

A Korean three-way comparison placed wild-simulated ginseng lowest—or tied lowest—for six ginsenosides (five majors and Rh1), below both 4- and 6-year field ginseng [19]. A Chinese metabolomic comparison of 13–20-year forest ginseng with 6-year garden ginseng found Rc, Rb1, Re, and Rf *lower* in forest material, while Rd and the rare ginsenosides Rg3, Rh1, and Rh2 were higher; the authors interpreted this as a shift in profile rather than a uniform increase [24]. For American ginseng, the direction was class-dependent: octillol-type ginsenosides were more abundant and diverse in wild roots, whereas the ginsenosides dominant in cultivated roots were elevated 2.6–14.4-fold over wild roots in normalized ion intensity [18].

The two genuinely wild *P. ginseng* roots quantified in the largest comparative dataset carried strikingly higher totals (50.5–55.5 mg/g of seven quantified ginsenosides) than either forest-grown or field samples [8], and wild roots also ranked highest in the Korean three-way comparison, whose means rest on five of seven roots [19]. With fewer than ten roots in two studies, these data cannot support any general statement about wild ginseng as a class, and we draw no conclusion about it.

The strength of the majority direction should not be overstated even where it holds. In that same dataset, forest-grown and field ranges overlapped almost completely (5.69–18.74 versus 2.99–19.55 mg/g, the field maximum exceeding the forest maximum), and only Re and Rf differed significantly between the two cultivated systems. Thus, “forest > field” describes group means rather than individual batches [8]. The discriminant thresholds of the largest recent comparison were likewise offered as descriptions that hold “in most cases” rather than as validated cut-offs [20].

Age-matched comparisons are few, and their results are mixed (Table 3). Only five studies in the corpus compared forest-based and field-cultivated ginseng at the same age. Neither of the two that quantified the major ginsenosides absolutely showed the uniform enrichment reported in many age-unmatched comparisons, and the one classed as forest higher rests on relative abundances in an untargeted analysis. In 2-year-old roots grown in the same county, 21 of 26 differential terpenoids were higher in forest-cultivated ginseng—including Rd and the rare ginsenosides Rh4, compound K, Rh2, F1, F2, and Rg3—but Rb1, Rg1, Re, and Rc did not meet the differential criteria [25]. In 5-year-old Korean roots, mountain-cultivated material carried a modestly higher total (18.01 versus 14.31 mg/g), yet field-cultivated ginseng contained more than twice as much Rg1 (3.25 versus 1.45 mg/g) and more Rf, while Rb1 was essentially equal [26]. In 5-year-old Chinese material, forest-simulating and greenhouse cultivation differed significantly only in Re, which was higher in the forest-simulating system, and the large advantage of the forest system appeared only when 15-year forest roots were compared with 5-year field roots [27].

The one age-matched study that did report a broad forest advantage—19 constituents, mainly PPD-type, at higher relative abundance in 15-year mountain-cultivated than in 15-year garden ginseng, with four constituents (mainly carbohydrates) higher in garden ginseng—was an untargeted comparison without absolute quantification [28]. It therefore established a compositional difference in favor of forest material rather than a content ranking. The largest age series, spanning 1–7 years in both systems from one region, found no separation between the two systems in whole-profile principal-component space until wild-simulated roots exceeded 7 years, after which separation rested on 54 discriminating ginsenosides, including four rare dehydrated ones (Rk3, Rh4, Rk1, Rg5) [12]. A lack of separation in principal-component space shows that the dominant axes of variation were shared, not that the profiles were identical; individual ginsenosides may still have differed. A Chinese survey spanning both variables showed the age crossover directly: summed contents of six ginsenosides rose with age in both systems and were higher in forest-cultivated ginseng at every age except the youngest forest class, which fell slightly below mature garden ginseng [29].

Taken together, these comparisons indicate that much of the apparent system effect on major ginsenosides may be attributable to, or substantially influenced by, cultivation age. At matched ages, the evidence does not support a uniform enrichment of major ginsenosides in forest-based material, but neither does it establish equivalence: the differences reported are mixed in direction [26], confined to a single ginsenoside [27], relative in nature [28], or carried by minor ginsenosides, deglycosylated or dehydrated [12,25].

**Table 2:** Direction tally of within-study contrasts between cultivation systems. Each row represents one study; values are as reported. The 18 *Panax ginseng* studies are listed first; the two *P. quinquefolius* studies, included as context only, are listed separately at the end. The class assigned in the direction tally (Section 5.1) is given in bold at the start of the “Reported direction” column.

Study	Systems Compared (Samples)	Ages Compared	What Was Compared	Reported Direction	Platform/Basis
Wang 2021 [8]	W (2) · F (27) · G (11), China	50–100 · 8–25 · ~6 years	7 Ginsenosides, sum; 177 saponins identified	**Forest higher.** W ≫ F ≳ G (F mean ~12.7, G ~9.1 mg/g); ranges overlap, only Re and Rf differ significantly F vs. G	HPLC-DAD, single-standard QAMS; absolute
Zhang 2025 [20]	F “MFCG” (20) · G (8–10), China	5–20 · 4–6 years	Rb1, most ginsenosides, PPD/PPT, Rb1/Ro	**Forest higher.** F > G (Rb1 6.61 vs. 3.17 mg/g; PPD/PPT ≈ 2 vs. ≈1); thresholds hold “in most cases”	HPLC-DAD; absolute (supplementary tables)
Qu 2023 [7]	WS “MCG” · G, 57 batches, China	Not matched	213 Ginsenosides quantified	**Forest higher.** Forest > G (“more abundant”); 25-year MCG distinct	2D-LC/Q-trap; absolute + quasi-quantification
Xu 2020 [30]	WS “MCG” · G, China	Not matched	20 Ginsenosides incl. rare	**Forest higher.** Forest ≈ 2× G; largest gap Rh2, Rh1	HPLC-UV; absolute
Li 2021 [28]	WS · G, China	15 · 15 years	23 Quality markers	**Forest higher.** 19 Constituents (mainly PPD) higher in forest	UPLC-QTOF; relative
Suh 2010 [31]	F (10 stands) · G, Korea	Not stated	Rb1, Re	**Forest higher.** Forest > G in all stands; regional variation	HPLC; values published only as a bar chart
He 2010 [29]	F · G, China	9–14 · 3–6 years	6 Ginsenosides, sum (15.2–54.7 mg/g); adenosine	**Forest higher.** Forest > G at every age except the 9-year forest class, which fell below garden ginseng; adenosine higher in G	RP-HPLC; absolute
Liu 2026 [27]	Forest-simulating · greenhouse, China	5 · 5 years and 15 · 5 years	Ginsenoside content + rhizosphere microbiome	**No simple ranking (Re only, age-matched).** Age-matched: only Re differed significantly (higher in forest-simulating roots); large forest advantage only for 15-year vs. 5-year	UPLC-MS/MS (MRM; 10 ginsenosides, external standards) + 16S; absolute
Zhu 2018 [32]	WS · G, China	12–20 · 4–6 years	126 Components, markers	**No simple ranking.** Composition similar; marker contents differ (age/system confounded)	UPLC-QTOF; relative
Wang 2016 [33]	F · G roots + rhizomes (16 samples), China	Incl. F 20–30 years	19 Ginsenosides	**No simple ranking.** Both quantified; organs pooled (Section 5.3)	HPLC-ESI-MS; absolute
Li 2018 [34]	F · G stem-leaf, China	Not matched	18 Ginsenosides; 26 markers	**No simple ranking.** Discriminable; direction not summarized	Orbitrap PRM; absolute (stem-leaf only)
Zeng 2026 [35]	G · transplanted · MCG, 30 batches, China	Not matched	Untargeted profile; immunomodulatory assay	**No simple ranking.** 7 Discriminatory biomarkers; MCG strongest activity; no ranking of majors	UPLC-Q-Orbitrap; relative
Yan 2023 [25]	F · G, same county, China	2 · 2 years	105 Terpenoids	**No simple ranking.** 21 of 26 differential compounds ↑ forest (Rd + rare Rh4, CK, Rh2, F1, F2, Rg3); Rb1, Rg1, Re, Rc not differential	UPLC-MS/MS; relative
Lee 2021 [26]	G (Geumsan) · MCG (Hamyang), Korea	5 · 5 years	Total and individual ginsenosides	**Mixed.** Total 18.01 (MCG) vs. 14.31 mg/g (G); Rg1 3.25 (G) vs. 1.45 (MCG)—field > 2×; Rf also higher in G; Rb1 equal	HPLC; absolute
Jeong 2010 [19]	W · G 4- and 6-year · WS, Korea	20–50 · 4/6 · 8–9 years	Rb1, Rc, Rd, Rf, Rg1, Rh1 (and Rb2, Re)	**Field higher.** W > G > WS (WS lowest or tied lowest) for six ginsenosides (five majors and Rh1)	HPLC-UV; absolute (W: means of 5 of 7 roots; basis possibly *n*-BuOH fraction)
Chen 2018 [24]	F “LXSS” (18) · G (6), China	13–20 · ~6 years	Untargeted profile	**Mixed.** Rc, Rb1, Re, Rf lower in forest; Rd, Rg3, Rh1, Rh2 higher—a profile shift, not a uniform increase	UPLC-Q-TOF-MS; relative
Zhu 2021 [36]	F “MFCG” · G, China	Not matched	10 Ginsenosides; PPD, PPT, total	**Forest higher.** F > G throughout (total 3.02 vs. 1.78% *w*/*w*; Rb1 1.03 vs. 0.37%)	HPLC; absolute (basis unstated)
Li 2025 [12]	WS (4 plants/age × 2 organs) · G farmland + G deforestation, Jilin	1–7 · 1–7 years (matched); 7–20 · 1–7 years; 10/13 · 10/13 years (rare markers only)	359 Ginsenosides; Rk3, Rh4, Rk1, Rg5 calibrated	**No simple ranking.** Age-matched 1–7 years: whole profiles not separated in PCA; ≥7-year WS separated by 54 compounds incl. 4 rare dehydrated; Rh4 41.5–49.7× and Rg5 17.6–21.0× in WS fibrous roots, ≥26.4×/≥11.7× at matched 10/13 years; direction of majors not reported	UHPLC-Orbitrap; relative (majors) + absolute (4 rare)
*Panax quinquefolius* (context only; not included in the *P. ginseng* tally)					
Liu 2022 [18]	*Panax quinquefolius* wild (12) · cultivated (19), USA/Canada	7–12 · 5 years	Untargeted profile	**Mixed (context).** Class-dependent: OT-type ↑ wild; dominant ginsenosides ↑ cultivated 2.6–14.4×	UHPLC-HRMS; relative
Liang 2019 [37]	*P. quinquefolius* wild (28) · cultivated (3), Tennessee	Not matched	10 Ginsenosides, sum	**Wild higher (context).** Wild mean 9.86 vs. 2.85 mg/g, but wild range 1.30–22.89 mg/g spans 17.6-fold; cultivated *n* = 3	HPLC; absolute, dry weight

W, wild; WS, wild-simulated; F, forest-cultivated; G, field (garden)-cultivated; MCG, mountain-cultivated ginseng; MFCG, mountainous forest-cultivated ginseng; LXSS, Lin-Xia-Shan-Shen (forest ginseng); HPLC, high-performance liquid chromatography; RP-HPLC, reversed-phase HPLC; UPLC, ultra-performance liquid chromatography; UHPLC, ultra-high-performance liquid chromatography; 2D-LC, two-dimensional liquid chromatography; DAD, diode array detection; UV, ultraviolet detection; MS, mass spectrometry; MS/MS, tandem mass spectrometry; ESI, electrospray ionization; QTOF or Q-TOF, quadrupole time-of-flight; Q-trap, hybrid triple quadrupole–linear ion trap; HRMS, high-resolution mass spectrometry; MRM, multiple reaction monitoring; PRM, parallel reaction monitoring; QAMS, quantitative analysis of multicomponents by a single marker; 16S, 16S rRNA gene sequencing; PPD, protopanaxadiol; PPT, protopanaxatriol; OT, octillol; CK, compound K; PCA, principal component analysis; *n*-BuOH, *n*-butanol; *n*, number of samples; ↑, higher in the system named after the arrow; ≫, much higher than; ≳, slightly higher than or similar to; ·, separates the systems or ages compared.

Four features of this tally matter more than the headline count. First, thirteen of the eighteen *P. ginseng* contrasts were not age-matched—in seven, forest material was older, typically 10–25 years against 4–6 years for field material (Section 5.2), and in six the ages compared were not stated or not matched—and the five age-matched comparisons gave mixed results, neither of the two with absolute quantification of the major ginsenosides showing a uniform enrichment in forest-based material. Second, the reversals cannot be explained away by method: Jeong 2010 [19] used one HPLC platform across all systems, and Lee 2021 [26] compared 5-year roots of both systems in a single laboratory. Third, where the majority direction holds, it reflects differences in group means with substantially overlapping ranges rather than a separation of populations. Fourth, apart from one higher total accompanied by lower Rg1 and Rf [26], the contrasts that survive age matching concern composition—minor (deglycosylated or dehydrated) ginsenosides, class ratios, multivariate fingerprints—rather than total content. For this reason, the record is best described as reporting differences rather than establishing a ranking.

Table 3 summarizes the five age-matched comparisons on the attributes that determine how much weight each can bear: analytical platform, absolute versus relative quantification, germplasm information, the exact age-matching conditions, and the reported profile shifts.

**Weight of evidence.** The conclusions drawn from the age-matched comparisons rest on a small and heterogeneous evidence base. Two of the five quantified the major ginsenosides absolutely [26,27]; one quantified four rare ginsenosides absolutely but compared the majors by relative peak area [12], and two relied on relative or untargeted measurements [25,28]. Each was conducted by a single research group, mostly within one region, sample sizes were small (for example, four plants per age class [12]), and none reported the germplasm of the compared material. Small single-group studies have limited power to detect modest differences, so false-negative results cannot be excluded. We therefore distinguish two statements: the current record provides no consistent evidence that forest-based cultivation raises major-ginsenoside concentrations once age is matched, but it does not demonstrate that no such difference exists. The absence of a consistent difference in five small comparisons should not be read as evidence of absence; adequately powered, age- and germplasm-matched studies with absolute quantification are required to settle the question (Section 12).

**Table 3:** Age-matched comparisons between forest-based and field-cultivated *Panax ginseng* (values and directions as reported).

Study	Systems, Location, Samples	Age Matching	Platform; Quantification	Germplasm	Reported Profile Shift
Li 2021 [28]	WS (MCG) · G; Huanren County, Liaoning, China; 12 batches	15 · 15 years	UHPLC/IM-QTOF-MS, untargeted; relative	Not reported	23 quality markers; 19 constituents (mainly PPD-type) at higher relative abundance in WS, 4 (mainly carbohydrates) higher in G; no absolute contents
Yan 2023 [25]	F · G; same county, China	2 · 2 years	UPLC-MS/MS; relative	Not reported	21 of 26 differential terpenoids higher in F (Rd and rare Rh4, CK, Rh2, F1, F2, Rg3); Rb1, Rg1, Re, Rc not differential
Lee 2021 [26]	MCG (Hamyang) · G (Geumsan), Korea	5 · 5 years; different sites	HPLC; absolute	Not reported	Total 18.01 (MCG) vs. 14.31 mg/g (G); Rg1 3.25 (G) vs. 1.45 mg/g (MCG); Rf higher in G; Rb1 similar
Liu 2026 [27]	Forest-simulating (mixed coniferous forest) · arched greenhouse; Ji’an, Jilin, China; *n* = 12 per group	5 · 5 years (also 15 · 5 years)	UPLC-MS/MS (MRM), 10 ginsenosides with external standards; absolute	Not reported	At 5 · 5 years only Re significantly higher in forest-simulating roots; Rb1 and Rg1 + Re numerically but not significantly higher; broad advantage only for 15- vs. 5-year roots
Li 2025 [12]	WS · G (farmland and deforested land); Jilin, China; 4 plants per age class, taproots and fibrous roots	1–7 · 1–7 years; 10/13 · 10/13 years (rare markers only)	UHPLC-Orbitrap MS; majors relative, 4 rare ginsenosides absolute	Not reported	No separation in whole-profile PCA at 1–7 years; at matched 10/13 years Rh4 ≥ 26.4× and Rg5 ≥ 11.7× higher in WS fibrous roots (µg/g scale)

WS, wild-simulated; MCG, mountain-cultivated ginseng; F, forest-cultivated; G, field (garden)-cultivated; HPLC, high-performance liquid chromatography; UPLC, ultra-performance liquid chromatography; UHPLC, ultra-high-performance liquid chromatography; MS, mass spectrometry; MS/MS, tandem mass spectrometry; IM-QTOF, ion mobility quadrupole time-of-flight; MRM, multiple reaction monitoring; PPD, protopanaxadiol; CK, compound K; PCA, principal component analysis; *n*, number of samples; ·, separates the systems or ages compared.

**Table 4:** Study-level quantitative anchors from full-text-verified studies (values as reported; recomputed only where the printed value is internally inconsistent, see Appendix A).

Contrast	Value	Caveat	Study
Under-forest vs. field, 7 quantified ginsenosides, root + rhizome	~12.7 vs. ~9.1 mg/g (group means, *n* = 27 vs. 11)	Recomputed from individual samples; QAMS; basis unstated	Wang 2021 [8]
MFCG vs. GG, whole root Rb1	6.61 vs. 3.17 mg/g	Supplementary tables, units confirmed against text; stated *n* inconsistent	Zhang 2025 [20]
MFCG vs. GG, whole root, sum of 7 quantified ginsenosides	24.9 vs. 20.7 mg/g (*n* = 20 vs. 8)	Same dataset, not age-matched (5 to ≥20 vs. 5 years); ~1.2-fold; group means as printed (Table S10), which include whole-root values computed from parts (Appendix A, item 6)	Zhang 2025 [20]
MFCG age classes, sum of 7 quantified ginsenosides, whole root	22.2 (5–7 years) → 24.9 (10–12) → 26.7 (15–17) → 25.6 mg/g (≥20 years); within-class range up to 1.7-fold	Recomputed from the three roots per class analyzed whole; 23.0 → 23.3 → 26.2 → 27.2 mg/g with the two part-derived roots per class included (Appendix A, item 6); ~1.2-fold range either way	Zhang 2025 [20]
Plant part within MFCG, sum of 7 quantified ginsenosides	Fibrous root 66.2 · rhizome 45.6 · lateral root 18.5 · main root 15.8 mg/g (*n* = 8)	4.2-fold gradient within one plant; GG shows the same order (44.6/33.9/25.1/14.0, *n* = 3)	Zhang 2025 [20]
Discriminant thresholds	Rb1 ≥ 6 mg/g (MFCG) vs. <6 (GG); Σ7 ≥ 25 mg/g for ≥10-year MFCG	Thresholds, not means; fewer than half of the individual whole roots aged ≥10 years reach the Σ7 value	Zhang 2025 [20]
WSG 14-year, mixed vs. coniferous stand	~53.5 vs. ~39.4 mg/g DW (21 ginsenosides)	Site contrast within one system; *n* = 5; direction per data (text contains a transposition)	Yun 2025 [38]
WSG 5-year, optimized extraction	Up to ~32.8 mg/g (15 species)	Extraction-condition dependent; basis unstated	Kim 2022 [39]
Age-matched field vs. mountain-cultivated, 5 years, total	14.31 (G) vs. 18.01 mg/g (MCG); Rg1 3.25 (G) vs. 1.45 (MCG)	One laboratory, one age; direction differs by ginsenoside	Lee 2021 [26]
MFCG vs. GG, whole root, total of 10 ginsenosides	3.02 vs. 1.78% *w*/*w* (1.70-fold); Rb1 1.03 vs. 0.37% (2.78-fold)	Basis unstated; sample number not reported	Zhu 2021 [36]
Wild vs. cultivated American ginseng, sum of 10	9.86 vs. 2.85 mg/g (3.46-fold)	Cultivated *n* = 3; dry weight	Liang 2019 [37]
Spread within a single wild population set	1.30–22.89 mg/g across 28 wild roots (17.6-fold)	Largest within-category spread in the charted record	Liang 2019 [37]
Cultivar effect under identical field culture	8.0 (Chunpoong) to 18.9 mg/g (Gopoong), five Korean cultivars	>2-Fold—the magnitude of the reported system effect	Ahn 2008 [40]
Rare ginsenosides Rh4, Rg5, aged WSG vs. GG, fibrous roots	Rh4 41.5–49.7× · Rg5 17.6–21.0× vs. two GG systems (1–7 years); ≥26.4× · ≥11.7× vs. 10/13-year GG	Absolute quantification of four rare ginsenosides only (µg/g scale); fibrous roots; *n* = 4 plants per age class; germplasm unreported	Li 2025 [12]
Commercial field soil, total ginsenosides (peak, 3.5 years)	8.4–34.6 nmol/g soil (*P. quinquefolius*)	recovery-adjusted; declines after year 4 (Section 8.3)	Rabas 2025 [41]

QAMS, quantitative analysis of multicomponents by a single marker; MFCG, mountainous forest-cultivated ginseng; GG, garden ginseng; G, field (garden)-cultivated ginseng; MCG, mountain-cultivated ginseng; WSG, wild-simulated ginseng; DW, dry weight; Σ7, sum of seven quantified ginsenosides; *n*, number of samples; →, next age class; ·, separates the values listed; ×, fold.

### 5.2. Cultivation Age

Age is the variable most confounded with system: forest-based roots are harvested at 7–30+ years, whereas field ginseng is typically harvested at 4–6 years. Thus, the system comparisons at unequal ages partly measure age. Within-system evidence indicates age effects that are real but neither linear nor universal. In the largest part- and age-resolved dataset, whole-root sums of seven ginsenosides quantified by the authors in forest-cultivated ginseng changed only modestly with age: class means were ~22.2, 24.9, 26.7, and 25.6 mg/g at 5–7, 10–12, 15–17, and ≥20 years for the three roots per class analyzed whole (Table 4), and ~23.0, 23.3, 26.2, and 27.2 mg/g when the two roots per class whose whole-root values were computed from their parts are included. Either way, the class means spanned only ~1.2-fold, less than the up to 1.7-fold spread among individual roots within a single class [20]. Elsewhere, correlations between stated age and individual ginsenosides across 40 samples were positive but heterogeneous [8]; the provisional ≥10-year threshold noted above rests on the same 5–20-year series, in which fewer than half of the individual whole roots aged ≥10 years reach 25 mg/g [20]; growth-age chemical change in mountain-cultivated ginseng is documented for 5–25-year material [42]; and metabolomic age-discrimination models succeed because age leaves multivariate rather than single-marker signatures [43,44], with rhizome-joint counts serving as a morphological proxy [45].

Korean wild-simulated series document that growth and functional properties vary with age between 3 and 9 years while morphology plateaus after 5 [46], and that growth traits and ginsenoside contents in 4-year-old material differ by region [47]. Two caveats recur: in wild and market material, age is self-reported or estimated (root scars, rhizome joints), with error that propagates into every age–content regression, so these should be read as associations rather than calibrations. Moreover, transcriptomic evidence suggests that age effects on biosynthesis may be smaller in wild than in cultivated plants [22], cautioning against transferring field-derived age curves to forest systems.

The most comprehensive age series available addresses both points directly. Profiling 359 ginsenosides by UHPLC-Orbitrap MS in garden ginseng aged 1–7 years (two field-type systems) and wild-simulated ginseng aged 1–7, 10, 13, 15, 17 and 20 years—four plants per age class from one region in Jilin, each age class a separate planting cohort harvested in the same October, with taproots and fibrous roots analyzed separately—the authors report that profiles stabilize at approximately 4 years in garden ginseng and 7 years in wild-simulated ginseng. Convergent evidence includes an Rg1/Re ratio crossing unity, optimal two-stage orthogonal partial least squares–discriminant analysis (OPLS-DA) segmentation, time-series clustering, and a fall in replicate-to-replicate variance with age, leading them to propose those ages as harvest optima [12]. The authors framed their study with the observation that same-aged wild-simulated and garden ginseng show negligible differences in total saponin content and in pharmacopoeial marker ginsenosides, and that most previous comparisons set older forest roots against young field roots [12].

Across the same age range (1–7 years) the two systems did not separate in whole-profile principal-component space, which does not by itself show that their profiles were identical; only wild-simulated roots of 7–20 years separated from garden ginseng, based on 54 discriminating ginsenosides that included four rare dehydrated compounds (Rk3, Rh4, Rk1, Rg5)—the only compounds in the study calibrated against standards. In fibrous roots of aged wild-simulated ginseng, mean Rh4 was 41.5–49.7-fold and mean Rg5 17.6–21.0-fold the level in the two garden-ginseng systems, with no overlap between the garden maximum and the wild-simulated minimum. In a supplementary comparison with 10- and 13-year-old garden ginseng, the same compounds remained at least 26.4- and 11.7-fold higher in wild-simulated fibrous roots. Thus, the rare-ginsenoside signature survives age matching, although the authors themselves concluded that reciprocal-planting experiments are still needed to separate age from environment, and the germplasm of the compared systems is not reported. Three features of this result matter for the present review: first, the system difference is carried by rare rather than major ginsenosides, whose contents were compared only by relative peak area, consistent with the age-matched comparisons above; second, the rare markers are quantified at the microgram-per-gram scale, two to three orders of magnitude below the major ginsenosides, so a fifty-fold ratio represents a small absolute mass; and third, the signature is organ-specific, meaning that a study sampling taproots and one sampling whole roots will not report the same system contrast.

Why rare dehydrated ginsenosides should accumulate in old forest roots is not known, but their chemistry constrains the candidate routes. Rk1 and Rg5 are C-20 dehydration products of the protopanaxadiol-type ginsenoside Rg3, and Rh4 and Rk3 are the corresponding products of the protopanaxatriol-type Rh1; in processed ginseng they form when heat and acidity first remove sugars and malonyl groups from the major ginsenosides and then dehydrate the side chain [48,49]. Three non-exclusive processes could contribute to them in living or freshly harvested roots. First, in vivo glycoside hydrolysis: a ginsenoside β-glucosidase has been purified from ginseng root [50], and endophytic bacteria from *P. ginseng* convert Rb1 to Rg3 [51], providing the deglycosylated precursors from which dehydrated products derive [14]. Second, malonylation and demalonylation: protopanaxadiol ginsenosides occur largely as labile malonyl-ginsenosides in fresh roots [49], so their turnover in planta or during drying changes the pool of neutral ginsenosides available for further conversion [52]. Third, progressive accumulation over many growing seasons in perennial tissues, notably the fibrous roots in which the signature was strongest [12]. The first two processes, however, act only on precursor supply—glycoside hydrolysis yields less glycosylated ginsenosides such as Rg3 and Rh1, whereas demalonylation yields neutral glycosides that still require deglycosylation—and no enzyme catalyzing the C-20 dehydration step has, to our knowledge, been demonstrated in living ginseng; because the step is favored by heat and acidity, the signature may also form during drying, storage, or extraction in proportion to a larger precursor pool in old fibrous roots. In LC–MS data, in-source loss of water from Rg3 or Rh1 can also produce ions of the same composition as the dehydrated products, so their identification requires chromatographic separation and comparison with reference standards; the four rare compounds in [12] were quantified against such standards. None of these processes has been tested in forest-grown roots, and because drying conditions alone alter malonyl-ginsenoside preservation [52], post-harvest artifacts must be excluded before the rare-ginsenoside signature is attributed to cultivation. The biological importance of this signature should also not be overestimated. The rare markers occur at microgram-per-gram levels, two to three orders of magnitude below the major ginsenosides; rare ginsenosides are, by definition, of low natural abundance, and high-quality clinical data on them remain inadequate [53]. We are not aware of dose–response data linking their concentrations in forest-grown roots to any pharmacological effect.

### 5.3. Plant Part

Most quantitative records concern root or root-plus-rhizome without separating the organs. The largest root/rhizome dataset in the corpus reports 19 ginsenosides across 16 samples but pools the organs by design (“roots and rhizomes”), so organ-resolved values cannot be recovered [33]. Where parts are resolved, differences are substantial: stem-leaf material carries a profile distinct from roots and discriminates forest from field ginseng on its own [34]. Supplementary data of the mountain-forest-cultivated ginseng versus garden ginseng comparison show a 4.2-fold gradient within one plant—fibrous roots ~66 mg/g, rhizome ~46, lateral roots ~18, main root ~16 mg/g (sum of seven quantified ginsenosides, *n* = 8)—with the same ordering in field ginseng [20]. The within-plant difference between main-root and fibrous-root values exceeds most between-system differences in Table 4, although the two measures are not strictly comparable.

Berry, leaf, and fine-root fractions are commercially relevant but thinly quantified in the forest-ginseng literature; leaf ginsenosides across growing years are reported mainly in the Chinese-language literature [54]. “Content of ginseng” without part specification is not a usable datum.

### 5.4. Site and Harvest Timing

Within one system and age, site alters the profile. The clearest controlled contrast is 14-year-old wild-simulated ginseng grown in coniferous versus mixed forest stands: 18 of 21 ginsenosides were higher in the mixed-forest site, with totals of ~53.5 versus ~39.4 mg/g dry weight (DW) [38]. Ecological correlation studies of forest-cultivated ginseng link climatic and soil variables to composition [55] and rhizosphere soil factors to origin traceability [56]; Korean programs report site-property/growth/ginsenoside correlation matrices [57,58]; and harvest-month effects on growth, ginsenosides (Rd, Rg3(S), F1 accumulating over the season), and broader metabolite profiles are documented for wild-simulated ginseng [59,60,61].

**Figure 3:**
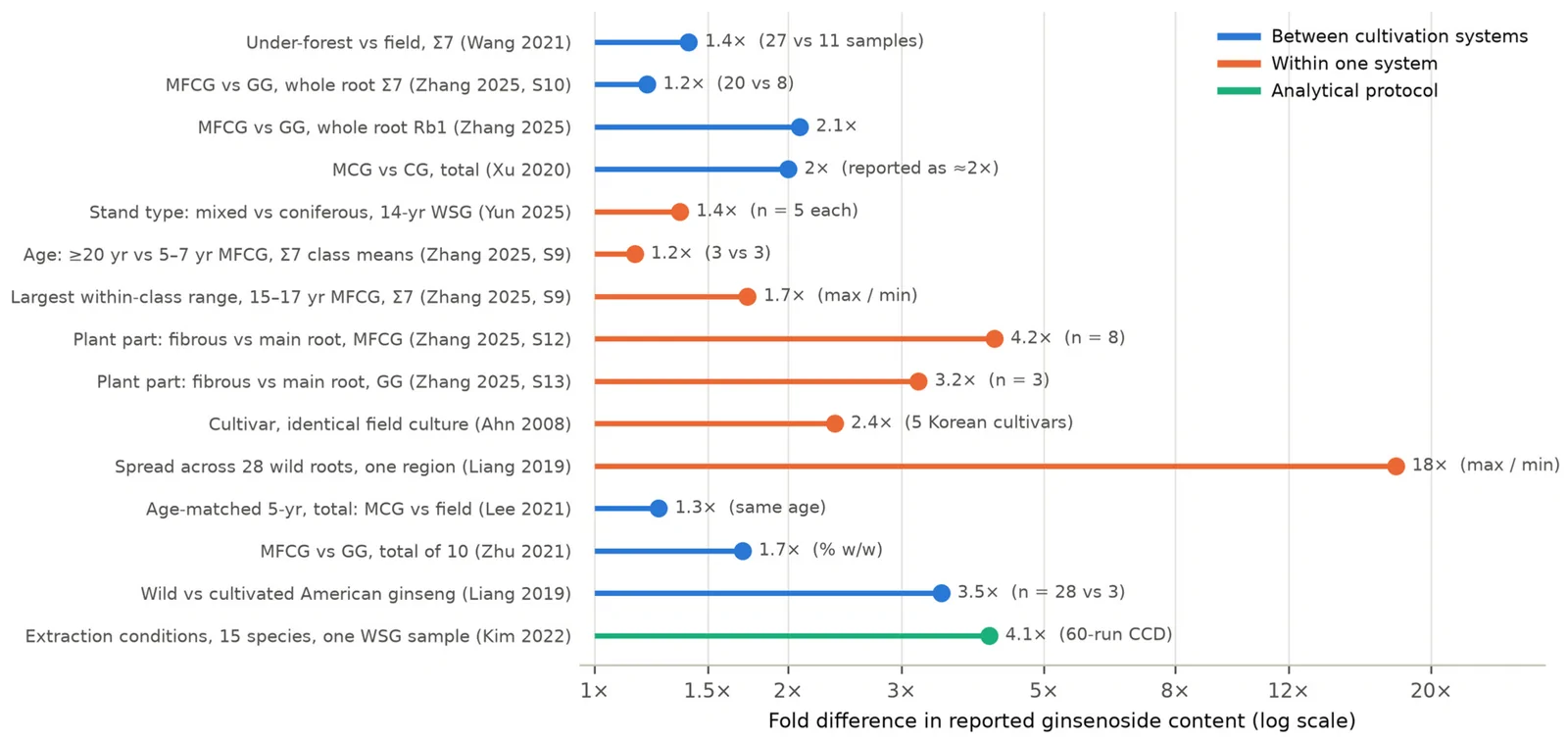
Magnitude of reported differences in ginsenoside content, expressed as fold ratios (log scale), for between-system contrasts (blue), within-system factors (orange), and analytical protocol (green). Each bar represents one study-level comparison as reported; values from Table 4 and the cited studies (Wang 2021 [8]; Zhang 2025 [20]; Xu 2020 [30]; Yun 2025 [38]; Ahn 2008 [40]; Liang 2019 [37]; Lee 2021 [26]; Zhu 2021 [36]; Kim 2022 [39]); S9, S10, S12, and S13 are supplementary tables of [20]. Because the bars combine different measures (ratios of group means, maximum-to-minimum ranges, within-plant organ gradients, and extraction effects), they illustrate the scale of variability rather than a formal comparison of effect sizes.

The widest spread in the charted record is not between systems but within one of them: across 28 wild American ginseng roots collected in Tennessee, the sum of ten ginsenosides ranged from 1.30 to 22.89 mg/g—a 17.6-fold spread of individual values rather than a ratio of means—while the same study’s wild-versus-cultivated difference was 3.46-fold [37]. Figure 3 places these magnitudes side by side. The measures differ in kind—ratios of group means, maximum-to-minimum ranges within a sample set, organ gradients within a plant, and extraction effects within a single sample—and are not directly comparable, so the figure illustrates the scale of variability that any system comparison must contend with rather than showing that a particular factor exceeds the cultivation-system effect. With that caveat, a site effect of ~1.36-fold on total ginsenosides is similar in scale to the 1.2–1.7-fold forest-versus-field differences in summed ginsenosides in Table 4 (up to 2.8-fold for Rb1 alone), while the plant-part gradient (4.2-fold) and the effect of extraction conditions (4-fold; Section 6) are numerically larger, and the spread among same-aged samples from one system (up to 1.7-fold) is comparable. Single-site, single-part system comparisons should therefore be interpreted cautiously, and stand type, soil, altitude, aspect, and plant part belong in the minimal reporting set (Section 12).

### 5.5. Germplasm: The Uncontrolled Variable

None of the comparative studies in Table 2 report the germplasm of the compared systems, and none controls for it; the system contrast therefore carries an unquantified genetic component. Three lines of evidence show that this is not a hypothetical concern.

First, the reported cultivar effect is of a magnitude comparable to the reported system effect. Grown under identical field culture, five registered Korean cultivars differed more than two-fold in root ginsenoside content, from 8.0 mg/g in Chunpoong—the most widely planted cultivar—to 18.9 mg/g in Gopoong [40]. This range is of the same magnitude as the forest-versus-field differences in Table 4. Thus, uncontrolled germplasm alone could generate, or erase, an apparent cultivation-system effect.

Second, the germplasm of both systems is heterogeneous, and in opposite ways. Twelve registered cultivars are recognized in South Korea and nine in China, with one Chinese cultivar bred specifically for forest cultivation [62]. The national germplasm collection is dominated by landraces (86.5% of 451 accessions [63]), but this describes the collection rather than commercial production, and the cultivar composition of field plantings has not, to our knowledge, been documented. On the forest side, simple sequence repeat (SSR) genotyping of 80 Korean wild-simulated roots from five regions with cultivar-discriminating markers revealed a very diverse genetic background, and the authors attributed farm-to-farm quality variation to the practice of establishing plantings from whatever cultivar or regional landrace seed was available [64]. Wild-simulated ginseng is thus not a distinct genetic pool, and the genetic make-up of the field-grown comparators is equally undocumented; the system contrast is therefore confounded with germplasm rather than merely uncontrolled for it.

Third, the experiment that would resolve this is feasible and has been done once, in the sister species. Reciprocal transplantation of roots from eight wild *P. quinquefolius* populations into two forest-garden sites, followed by re-assay after two years, separated genotype from environment at the level of individual ginsenosides: Re was governed by population and not site, Rb1, Rc and Rb2 by site and not population, and Rg1 and Rd by both [65]. No equivalent design exists for *P. ginseng*; allele-specific and random amplified polymorphic DNA (RAPD) marker systems are available [66,67] but have not been paired with content data in a system comparison. Until they are, “forest-based systems produce different profiles” cannot be separated from “forest-based systems are planted with different plants”. We regard this as the single largest unaddressed confounder in the record and place it first among the design recommendations of Section 12.

Because paired, multi-year field trials are logistically demanding, three more tractable strategies deserve priority. First, germplasm can be documented retrospectively: SSR and SNP marker sets that discriminate Korean cultivars and germplasm [63,64,68,69] can be applied post hoc to harvested roots, provided that voucher tissue is retained, so that studies can report, and later stratify by, the genetic background of each sample. Second, common-garden designs can draw on existing germplasm collections in Korea and China [62,63]: seed of documented accessions sown at the same time under forest and field conditions—a split-seed-lot design—separates cultivation system from genotype without waiting for a reciprocal transplant of mature roots. Third, for the existing literature, sensitivity analyses can use cultivar or geographic seed provenance as a proxy, for example by repeating the direction tally after excluding studies of market-sourced material or by stratifying contrasts by region of origin. These steps would turn germplasm from a caveat into a measurable covariate.

### 5.6. From Ginsenoside Profiles to Pharmacological Differences

The price gap between forest-based and field-cultivated ginseng is commonly justified by an assumed difference in pharmacological efficacy, and it is natural to ask whether the ginsenoside differences charted above can account for it. On the current record, they cannot be shown to do so. A few studies have compared bioactivity directly: an integrative-pharmacology study of 15-year-old mountain- and garden-cultivated ginseng linked 23 differential constituents to predicted targets [28]; a multi-habitat comparison reported the strongest immunomodulatory activity for mountain-cultivated ginseng [35]; and extracts of mountain ginseng showed stronger anticancer activity against breast cancer and stronger anti-angiogenic activity than extracts of farm-cultivated ginseng [70,71]. None of these studies, however, matched cultivation age and germplasm, and none tested whether the activity differences were reproduced by the differentially abundant ginsenosides at the concentrations present in the roots. Whether profile differences translate into pharmacological differences therefore remains an open question rather than an established link.

Because individual ginsenosides differ in activity [1], monomer-level differences matter more than totals. Across the contrasts in Table 2, the direction for individual major ginsenosides is not consistent: Rb1 was reported higher in forest material in some comparisons [20,36] but lower [24] or similar [26] in others; Rg1 and Rf were higher in field ginseng in an age-matched absolute comparison [26]; and in the largest Chinese dataset only Re and Rf differed significantly between forest-grown and field samples [8]. Class ratios (PPD/PPT) and minor or rare ginsenosides—Rg3, Rh1, Rh2, and the dehydrated Rk3, Rh4, Rk1, and Rg5—are the features most consistently reported as higher in forest-based material [12,24,25,30], although not in every comparison [19]. These rare ginsenosides are therefore the most plausible chemical correlates of any cultivation-system difference in activity, but at present they are correlates only: their low concentrations and the absence of dose–response data (Section 5.2) mean that they cannot yet be regarded as the chemical basis of a pharmacological divergence.

Ginsenosides are also not the only bioactive constituents of ginseng. Polysaccharides, with immunoregulatory, antitumor, antioxidant, and hypoglycemic activities [72], and phenolic compounds such as chlorogenic and coumaric acids, whose content correlates with antioxidant activity [73], contribute to the reported activities of ginseng, and several comparisons in the corpus already show system differences beyond the ginsenosides: oligosaccharides together with ginsenosides discriminated forest-cultivated from garden ginseng [36], adenosine was higher in garden than in forest ginseng [29], organic acids and calcium oxalate crystals entered the discrimination criteria of a recent comparison [20], and nutritional constituents and antioxidant activity differed between cultivated and mountain-cultivated ginseng [26]. These data were not charted systematically here, because the search was built around ginsenosides. An integrative analysis that charts polysaccharides, phenolic acids, and other metabolite classes with the same attention to age, germplasm, and plant part is a natural extension of this review and is needed before efficacy differences are attributed to any single constituent class.

## 6. Why Published Values Resist Comparison

Four methodological axes fragment the quantitative record.

**Analytical platform and calibration.** The corpus spans HPLC-UV (203 nm), HPLC with evaporative light-scattering detection/charged aerosol detection (HPLC-ELSD/CAD), UPLC with photodiode array detection (UPLC-PDA), LC–MS/MS multiple reaction monitoring (MRM), 2D-LC, and untargeted quadrupole time-of-flight (QTOF)/Orbitrap platforms. Panel sizes range from 2 to more than 200 standards, and at least one major dataset calibrates one compound directly and derives six others through relative correction factors [8]. Charged-aerosol methods for polar and non-polar ginsenosides in wild-simulated ginseng appear mainly in the Korean literature [74]. Colorimetric “total saponin” assays are not ginsenoside-specific and cannot be pooled with chromatographic sums [75].

**Extraction and expression basis.** Extraction optimization demonstrates 4-fold swings in recovered totals within one sample across solvent, temperature, and time [30,39,76]. Papers frequently leave the denominator undefined (dry weight, fresh weight, or extract basis), and in one case, print no units at all in the supplementary content tables (Appendix A). A reported mg/g without extraction conditions and basis is at best a within-study comparator.

**Sample identity.** System labels are unverified in market-sourced material (Section 3), ages are self-reported, parts are pooled, germplasm is unreported (Section 5.5), and disease status is rarely stated, although red-rust-infected roots differ chemically from healthy ones [77].

**Reporting completeness.** Replicate structure (biological *n* versus injection replicates) and dispersion type (standard deviation versus standard error, sometimes unlabeled) are inconsistently reported. Appendix A lists the arithmetic and labeling inconsistencies identified in published tables. A distinct and surprisingly common failure is that the measurement is made but never printed: in three of the studies we retrieved in full, contents were quantified against reference standards and then presented only as unlabeled bar charts or as fold changes. Thus, the values exist in the literature in a form that no synthesis can use. None of these problems is individually prohibitive; however, taken together, they explain why this review reports direction tallies and study-level anchors rather than a pooled estimate.

## 7. Associated Environmental and Agronomic Factors

The site effects described in Section 5.4 have proximate agronomic correlates. Soil physicochemistry is the best studied: Korean surveys characterize the soil envelopes of successful wild-simulated and mountain-cultivated sites [78,79], and site and soil correlates of growth and ginsenoside accumulation in forest-grown ginseng are now modeled directly [80]. Stand composition (coniferous versus mixed) shifts the microclimate—air temperature, soil moisture, and solar radiation were all higher in the mixed stand—together with soil organic matter, nitrogen, and exchangeable cations, with the profile consequences reported above [38]. Planting method and stand management are associated with growth and saponin content [81]. At the intensive end, containerized non-agrochemical cultivation of 6-year ginseng reports profile consequences of its own [82].

What is conspicuously missing are factorial or longitudinal designs that vary one factor at a time in forest settings; nearly all current evidence is correlational across sites, with stand, soil, microbiome, age, and germplasm covarying.

## 8. The Root-Associated Microbiome: Associations and Candidate Mechanisms

**Figure 4:**
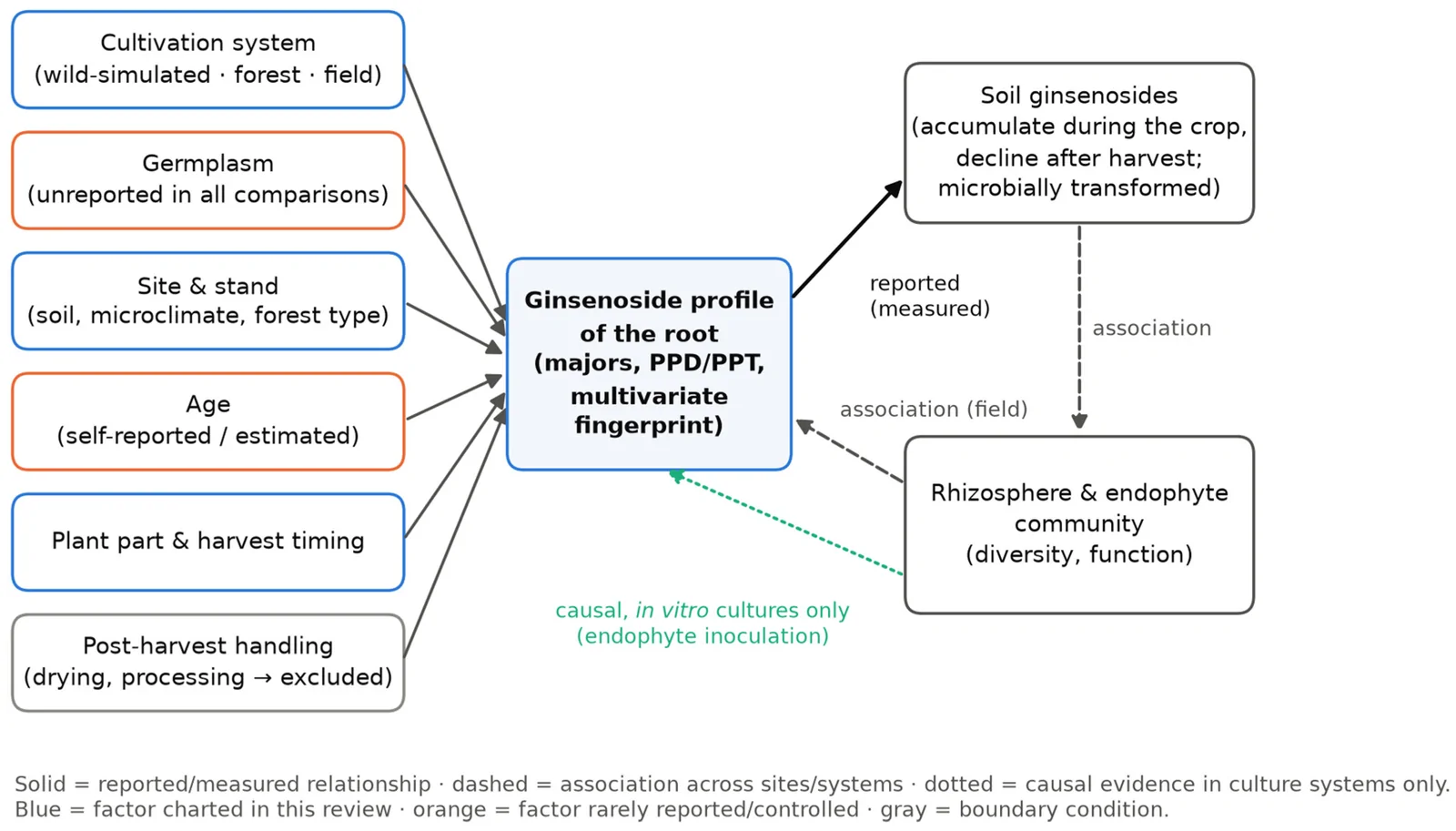
Factors associated with the ginsenoside profile of *Panax ginseng* roots and feedback through soil, as supported by the charted literature. Solid arrows indicate measured relationships (root ginsenosides enter the soil, accumulate during cultivation, and decline after harvest); dashed arrows represent associations observed across sites and systems; dotted arrows denote causal evidence available only from in vitro culture systems (e.g., endophyte inoculation of adventitious roots). Orange boxes highlight factors rarely reported or controlled in comparative studies. The mechanism linking soil ginsenosides to the replant problem remains contested (Section 8.3).

Figure 4 summarizes how the factors described in Section 5, Section 6 and Section 7, together with the root-associated microbiome, relate to the ginsenoside profile and indicates the level of evidence currently supporting each link.

### 8.1. Community Differences Track Cultivation System

Rhizosphere communities of wild and cultivated ginseng differ in both diversity and structure [83,84], as do communities across cultivation ages and modes [85,86,87]. Metagenomic studies extend these contrasts to function: forest-soil communities associated with wild-simulated ginseng carry distinctive nutrient-cycling and plant-interaction gene repertoires [88]. Single-study integrations now link rhizosphere composition, soil properties, and ginsenoside content within one design [9,10,11,89,90]. These remain associations across sites and systems; none yet separates microbiome from the co-varying factors described in Section 7. Whether community differences drive profile differences, or are themselves by-products of the soil physicochemical properties, stand, and age with which they covary, cannot be determined from these designs; they are reported here as field correlations, not causal findings.

### 8.2. Endophytes and Ginsenoside Accumulation

Mountain-cultivated ginseng hosts diverse culturable bacterial and fungal endophytes [91,92,93], some with demonstrated biocontrol activity [94,95,96,97]. Causal evidence that microbes enhance ginsenoside output currently comes only from in vitro culture systems: inoculating adventitious-root cultures with ginseng endophytic bacteria increased ginsenoside accumulation [98], and a saponin-producing endophytic fungus from wild ginseng produced saponins during fermentation [75]. Field translation has commenced—plant-growth-promoting rhizobacteria isolated from wild-simulated ginseng have been evaluated in application trials [99]—but no study has yet demonstrated microbiome-mediated profile change in forest-grown roots under controlled inoculation. We regard this as the pivotal missing experiment. Mycorrhizal colonization of wild ginseng was described two decades ago and remains almost unstudied quantitatively [100].

### 8.3. Ginsenosides in Soil: Ecological Roles and Mechanistic Debate

Root ginsenosides also enter the soil. In commercial *P. quinquefolius* gardens, total soil ginsenosides accumulated through the rotation, peaked at 8.4–34.6 nmol/g soil in the third growing season, and had largely disappeared after year four, which the authors attribute to microbial biotransformation [41]; equivalent field data for *P. ginseng* are not available. Ginsenoside-mediated autotoxicity and the replant problem have recently been reviewed in detail [101]. What remains contested is what sustains replant failure once parent ginsenosides have disappeared: a ginsenoside-selected microbial community and soil state [101]; a microbially derived post-harvest factor that increases susceptibility to root rot [102], with ginsenosides promoting the virulence of *Ilyonectria mors-panacis* [103]; or transformation of parent ginsenosides into more phytotoxic rare ginsenosides [104]. Interventions that reshape the rhizosphere—crop rotation, fallowing, and microbial management—alleviate replant failure regardless of which mechanism predominates [105,106,107,108]. Most of this evidence comes from laboratory and pot experiments.

Autotoxicity and replant disease are agronomic problems distinct from the forest-versus-field quality comparison, and their relevance to grading is indirect. Soil and microbiome factors matter for commercial grading only insofar as they change the ginsenoside profile of the harvested root, and that link has so far been shown only as field correlations across sites and systems (Section 8.1); causal evidence of microbial effects on ginsenoside accumulation is restricted to in vitro inoculation of root cultures [98]. Until inoculation or exclusion experiments in forest settings report profile endpoints, soil and microbiome data cannot serve as grading criteria; their practical value for growers lies in site selection and replant management (Section 11).

## 9. Processing and in Vitro Systems: Boundary Conditions

Values from steamed (red), fermented, or enzyme-converted material describe products rather than production systems and were excluded from all system comparisons above. Within a single wild-simulated lot, drying, steaming, and lactic fermentation reshaped the ginsenoside panel stepwise [109]; steaming temperature alone redirected composition in cultivated wild ginseng [110]; and wild-simulated versus field ginseng responded differently to steaming and fermentation [111,112]. Processed-ginseng biomarker panels are being standardized for precisely this reason [113]. An extensive bioconversion literature documents microbial and enzymatic conversion of Rb1 to minor ginsenosides [14,114,115]. Adventitious-root and cambial-cell cultures of ginseng, including lines derived from wild or mountain ginseng, are biotechnological products with their own quality logic [116,117]; their values were charted but treated separately. Callus and cell-suspension cultures differ further: despite substantial ginsenoside contents, some show narrower detected profiles than field-grown roots—suspension cultures of *P. ginseng* produced mainly Rb1 and Rg1 [118], and callus of *P. quinquefolius* contained four of the ten ginsenosides found in native roots [119]—so such biomass is not necessarily chemically equivalent to wild-simulated or field-cultivated roots and, on this compositional evidence, is a less promising substitute for whole-root preparations. These observations concern callus and suspension biomass; adventitious-root cultures were not compared with field- or forest-grown roots in this review, and no culture type should be presented as a substitute for whole roots without compositional and functional evidence. Even for “raw” comparisons, drying method matters: freeze- versus heat-drying alters malonyl-ginsenoside preservation, making post-harvest handling part of the profile [52].

## 10. Authentication, Standards, and the Industry Interface

Because system labels carry large price premia, discrimination methods are an active front: UPLC/orthogonal acceleration–TOF fingerprints separate mountain-cultivated from field ginseng [21]; untargeted metabolomics distinguishes mountain- from garden-cultivated material [32]; proteomic peptide markers authenticate mountain-cultivated versus cultivated ginseng [120]; calcium-oxalate, organic-acid, and ginsenoside-ratio thresholds have been jointly proposed as system-discrimination criteria [20]; and enhanced profiling frameworks combine targeted panels with chemometrics [7]. The practical gap lies in regulation: current quality standards specify only a small number of marker ginsenosides and moisture/ash limits, which neither capture system-discriminating features nor constrain the reporting deficiencies identified in Section 6.

## 11. Implications for Forest-Farming Practice

Read as agronomy rather than chemistry, the record supports a small number of practical statements, each bounded by its evidential limit:

- **Site selection as a quality lever.** Within one system and age, a mixed stand—warmer, moister, higher in organic matter and exchangeable cations—yielded ~1.36-fold higher total ginsenosides than a coniferous stand [38]. Because this rests on a two-site comparison that cannot separate stand composition from other site differences (altitude, aspect, soil, microclimate, and management), recommendations about stand type should remain provisional. Producers may reasonably record stand composition and soil chemistry when choosing among candidate sites, but replicated multi-site comparisons are needed before stand type can be recommended as a quality lever.
- **Age claims require verification.** Ginsenoside contents need not rise steadily with age. Thresholds such as a seven-ginsenoside sum ≥ 25 mg/g at ≥10 years [20] should be treated as provisional: in that study’s supplementary data, fewer than half of the individual whole roots aged ≥10 years reach the 25 mg/g value. Producers should record planting year and retain rhizome material for joint-count or marker-based verification [45].
- **Harvest timing and post-harvest handling matter.** Both measurably shift the profile [52,59]. Documenting harvest month and drying method costs little and preserves the value of later analysis.
- **Soil stewardship links quality to sustainability.** Ginsenosides accumulate in soil during rotation and are microbially transformed rather than simply persisting [41,104]. The replant problem they are implicated in remains mechanistically contested (Section 8.3). Site rotation, fallowing, and rhizosphere management are therefore integral to the quality system, not merely yield decisions.
- **Discriminability is better supported than superiority.** The literature supports “forest-grown roots are chemically distinguishable” far more strongly than “forest-grown roots contain more ginsenosides.” Given the reversals and heterogeneity documented above, claims framed on discriminability rest on firmer evidence than claims of superior ginsenoside content.

## 12. Research Gaps and Reporting Recommendations

Six gaps emerge from the charted record: (1) **Germplasm control**: comparative studies should report, and where possible match, the genetic source of compared systems. The reciprocal-transplant design already applied to *P. quinquefolius* [65] shows that a genotype-by-environment test is practicable and could resolve in one experiment what the observational record cannot; post hoc genotyping and common-garden designs offer more tractable alternatives (Section 5.5). (2) **Age matching**: the five age-matched comparisons available give mixed results, and neither of the two with absolute quantification of the major ginsenosides shows a uniform enrichment in forest-based material (Section 5.1, Table 3). Age-unmatched contrasts should therefore no longer be reported as system effects; where matching is impossible, age should be recorded and modeled, bearing in mind that statistical adjustment cannot separate age from system when their age ranges do not overlap. (3) **Site–system confounding**: multi-site designs that cross system with site, or at minimum report site covariates, are rare. Single-site system contrasts should be treated as provisional. (4) **Causal microbiome evidence**: inoculation or exclusion experiments in forest settings with profile endpoints do not yet exist (Section 8.2). (5) **Bilingual curation**: 62% of the retained records were retrieved outside the PubMed queries, and the Korean and Chinese literatures hold a substantial share of the primary measurements—including the age-matched comparisons that most constrain the field’s central claim. Monolingual, PubMed-only syntheses risk mischaracterizing the record, and systematic searches of CNKI, Wanfang, VIP, and the Korea Citation Index are a priority for future syntheses (Section 2.3). (6) **Linking chemistry to efficacy**: no study has tested whether ginsenoside differences between systems account for differences in bioactivity at matched age and germplasm, and non-ginsenoside constituents remain uncharted (Section 5.6).

As a recommendation rather than a standard, we suggest that ginsenoside content studies report the items in Table 5. None of these items is novel; their value lies in the fact that no study in Table 2 satisfies all of them.

## 13. Conclusions

The published quantitative record on wild-simulated and forest-grown *P. ginseng*, read at the level of studies rather than isolated values, supports four statements.

First, forest-based grades produce ginsenoside profiles that most within-study comparisons report as distinct from field-cultivated ginseng—in marker thresholds, class ratios, and multivariate fingerprints, and often with higher major-ginsenoside contents in forest-grown roots. However, the direction is not unanimous, and the five comparisons that hold cultivation age constant give mixed results: neither of the two that quantified the major ginsenosides absolutely shows a uniform enrichment of them, and, apart from one higher total accompanied by lower Rg1 and Rf, the differences that survive age matching are compositional, carried notably by minor (deglycosylated or dehydrated) ginsenosides in specific organs. This indicates a lack of consistent evidence for higher major-ginsenoside contents in forest-grown material, not proof that no difference exists.

Second, no consistent ranking is supported once age, site, plant part, and germplasm—which remain largely uncontrolled in the primary studies—are considered, and methodological heterogeneity further limits the validity of cross-study ranking. Within-system variation associated with those same factors appears comparable in scale to the reported between-system differences, although these measures are not strictly comparable. This is why single-value claims about the content of “wild-simulated” or “forest” ginseng are not defensible, and why no conclusion can be drawn about genuinely wild ginseng, whose quantitative record comprises fewer than ten roots in two studies.

Third, the emerging environmental and microbiome literature offers candidate mechanisms and a feedback loop through soil ginsenosides, but the decisive experiments—controlled germplasm, factorial site manipulation, and inoculation with profile endpoints in forest systems—remain to be done.

Fourth, whether the reported profile differences account for differences in pharmacological efficacy has not been tested: no study has linked bioactivity to specific ginsenosides at matched age and germplasm, the rare ginsenosides that best discriminate the systems occur at microgram-per-gram levels without dose–response data, and other constituent classes such as polysaccharides and phenolic acids have barely been compared between systems (Section 5.6).

For growers of wild-simulated and forest-grown ginseng, the practical message is that the quality they are paid for is an outcome of decisions they control—site and stand, verified age, which organ is sold, harvest timing and drying, and the seed they plant—rather than of the system label itself. The next decade of measurements will accumulate into evidence only if they are reported in a form that allows comparison. These conclusions are bounded by the search (Section 2.3): they describe the record retrievable through PubMed, Crossref, and hand-searching rather than the complete Chinese- and Korean-language literature.

## Figures and Tables

**Table 1:** Cultivation systems of *Panax ginseng* as used in this review, with synonyms encountered in the corpus.

System (This Review)	Synonyms in the Literature	Defining Features
Wild (W)	Wild ginseng, natural ginseng, sansam	Self-propagated in natural forest; age estimated; very few quantified samples available
Wild-simulated (WS)	Wild-simulated ginseng (WSG), mountain-cultivated ginseng (MCG), sanyangsam, cultivated wild ginseng	Sown or planted in forest sites; no agrochemicals; minimal management; typically harvested after ≥7 years
Forest-cultivated (F)	Forest ginseng (lín xià shēn), ginseng under the forest, mountainous forest-cultivated ginseng (MFCG)	Cultivated in forest sites with variable management intensity
Field-cultivated (G)	Garden ginseng, cultivated ginseng, field ginseng	Cultivated under artificial shade in raised beds; agrochemical inputs applied; typically harvested on a 4–6-year rotation

**Table 5:** Minimum reporting items for ginsenoside content studies of *Panax ginseng* and the comparability problems they address (Section 6).

Item	What to Report	Problem Addressed
Cultivation system	System per Table 1 terminology, with management practices described	Terminology confusion; unverifiable system labels
Germplasm	Seed or seedling source; cultivar or landrace if known	Uncontrolled genetic confounding (Section 5.5)
Site	Location, altitude, aspect, stand type, soil class	Site effects of the same order as system effects
Age	Verified or estimated age and method of estimation	Self-reported age propagates into age–content regressions
Plant part	Organs analyzed separately; pooling stated explicitly	Four-fold within-plant gradients
Harvest and handling	Harvest date; drying method; any processing	Seasonal and post-harvest shifts
Extraction	Solvent, temperature, time, solid-to-liquid ratio, cycles	Four-fold protocol dependence
Analysis	Platform, column, detection; list of standards; calibration mode (external vs. single-standard/quantitative analysis of multicomponents by a single marker (QAMS))	Platform and calibration heterogeneity
Expression basis	Dry weight, fresh weight, or extract basis, stated in every table	Undefined denominators
Replication	Biological *n*, injection replicates, dispersion type (standard deviation/standard error)	Unlabeled or ambiguous dispersion
Data	Individual-sample values in machine-readable supplements	Enables study-level re-synthesis

## Data Availability

The charted dataset (Table S1; 4199 values in a 19-field schema, with a data dictionary, source references, quality flags, and the reason each value was or was not used) is provided as Supplementary Materials and will be deposited in Zenodo upon acceptance. The complete search strings are provided in Table S2.

## Supplementary Materials

The following supporting information can be downloaded at: https://www.mdpi.com/article/10.3390/plants15213472/s1, Figure S1: Composition of the scoping corpus (264 unique studies; the 270 retained records include six pairs merged at the charting stage because they shared a DOI or a near-identical title). (a) Publication year by retrieval route; 61% of the 264 studies were not retrieved by the PubMed queries. (b) Topic categories used for charting; each study is assigned to one category here (chemistry × microbiome denotes studies charted in both); Table S1: The charted dataset (Excel workbook; 4199 reported values in a 19-field schema, with source references, quality flags, the reason each value was or was not used in natural-profile statements, and a data dictionary); Table S2: Complete search strategies for the four search arms, with search dates and numbers of records; Table S3: Completed PRISMA-ScR checklist.

## Appendix A. Internal Inconsistencies Detected in Published Content Tables During Charting

**Table A1:** Internal inconsistencies detected in published content tables during charting and their handling in this review.

#	Study	Observation	Handling in This Review
1	Wang 2021 [8], Table 4	Printed F-GS total (14.21 ± 10.85 mg/g) does not equal the sum of the printed component means (12.69) nor the mean of the 27 individual-sample totals in Table 3 (12.69 ± 4.07); the C-GS row reconciles exactly	Group mean recomputed from individual samples (Table 4 of this review)
2	Yun 2025 [38], §2.4	Text states coniferous > mixed by ~1.3×; the reported values (39.4 vs. 53.5 g/kg) and the remainder of the paper indicate mixed > coniferous	Direction taken from the data
3	Wang 2016 [33], Table 6	Ra1 entries for samples 14–16 (forest 20/25/30 years) duplicate those of samples 7–9, including dispersion; row sums for 14–16 do not reconcile	Ra1 rows for samples 14–16 excluded from the natural-profile subset (natural_profile = N in Table S1); printed totals retained as reported and flagged
4	Lee 2020 [17], Table 2 vs. abstract	Table totals are exactly 10× the abstract values; sample described as “wild” is 4-year-old market material described elsewhere as cultivated	Values not used for system comparison
5	Zhang 2011 [117], Table 4	Bioreactor CL4 printed total (4.22) differs from component sum (4.023)	Component values used; culture material excluded
6	Zhang 2025 [20], Table S10	GG column reproduces exactly from 8 of the 10 GG samples in Table S9 (two 9-year samples omitted), whereas text states *n* = 7; last 11 whole-root rows of Table S9 are composites of part values	*n* reported as reconstructed; composite rows excluded from the recomputed age-class means, with means including them given for comparison (Table 4 of this review); printed group means, which include them, quoted unchanged
7	Guo 2024 [113], Table S4	No units printed anywhere in the supplement; internal ratios exclude µg/g	Relative comparisons only; absolute values not compared across studies
8	Tajudeen 2023 [121], Table 6	Untreated control egg yolk reports 1.17–1.22 mg/g “ginsenoside” by gravimetric *n*-BuOH residue without standards; sample description inconsistent between Introduction and Methods	Excluded from all content statements
9	Zhu 2023 [14], Table 2	Values are substrate loads of in vitro enzyme reactions compiled from other studies, not sample contents	Charted as conversion data only
10	Yang 2021 [122]	Substrate is a commercial ginseng extract of unstated cultivation origin; link to wild ginseng is via soil-isolate screening only	Excluded from system comparisons
11	Liang 2019 [37]	Five statements in the text are not reproduced by the paper’s own Table 3: Rg2 is stated to occur in only two samples but is also tabulated at 0.11 mg/g in a third; the same value is described as “less than 0.11”; a stated ratio of “more than 3.5 times” recomputes to 3.46; a stated mean of 9.7 mg/g is not recovered from the table (28 wild samples give 9.86, all 31 give 9.18); and a stated Rb2/Rc mean of 3.86 with a minimum of 2.3 recomputes to 3.91 and 2.43	Table values used throughout; text-only figures not cited
12	Suh 2010 [31]	Rb1 and Re contents are presented only as an unlabeled bar chart, so no value can be transcribed, although the study quantified by HPLC; the abstract also refers to “10 other forests” where the design has ten stands in total	Direction cited; no value cited
13	Fang 2024 [89]	Eleven reference standards are specified, and quantification is described, but no absolute content appears anywhere in the text or tables—only fold changes and percentages reported	Excluded from quantitative charting
14	Li 2025 [12]	The stabilization age for wild-simulated ginseng is given as six years in Results and seven years in the Abstract, Discussion and Conclusion; the 49.73-fold figure, obtained against 1–7-year garden ginseng, is described in the Discussion as age-matched, whereas the age-matched value is ≥26.4-fold; the group size of the supplementary 10- and 13-year garden material is not stated	Seven years used (authors’ final conclusion); age-matched ratios quoted separately from the 1–7-year ratios

## References

[1] Ratan Z.A., Haidere M.F., Hong Y.H., Park S.H., Lee J.-O., Lee J., Cho J.Y. (2021). Pharmacological potential of ginseng and its major component ginsenosides. J. Ginseng Res..

[2] Liu L., Xu F.-R., Wang Y.-Z. (2020). Traditional uses, chemical diversity and biological activities of *Panax* L. (Araliaceae): A review. J. Ethnopharmacol..

[3] Yang W.-Z., Hu Y., Wu W.-Y., Ye M., Guo D.-A. (2014). Saponins in the genus *Panax* L. (Araliaceae): A systematic review of their chemical diversity. Phytochemistry.

[4] Nguyen N.-H., Ha T.K.Q., Yang J.-L., Pham H.T.T., Oh W.K. (2021). Triterpenoids from the genus *Gynostemma*: Chemistry and pharmacological activities. J. Ethnopharmacol..

[5] Shin B.-K., Kwon S.W., Park J.H. (2015). Chemical diversity of ginseng saponins from *Panax ginseng*. J. Ginseng Res..

[6] Shin S., Park M.S., Lee H., Lee S., Lee H., Kim T.H., Kim H.J. (2021). Global trends in research on wild-simulated ginseng: Quo vadis?. Forests.

[7] Qu H., Wang J., Yao C., Wei X., Wu Y., Cheng M., He X., Li J., Wei W., Zhang J. (2023). Enhanced profiling and quantification of ginsenosides from mountain-cultivated ginseng and comparison with garden-cultivated ginseng. J. Chromatogr. A.

[8] Wang C.Q., Yi L.W., Zhao L., Zhou Y.Z., Guo F., Huo Y.S., Zhao D.Q., Xu F., Wang X., Cai S.Q. (2021). 177 saponins, including 11 new compounds in wild ginseng tentatively identified via HPLC-IT-TOF-MSn, and differences among wild ginseng, ginseng under forest, and cultivated ginseng. Molecules.

[9] Liang Z.W., Guan Y.H., Lv Z., Yang S.C., Zhao M., Chen J.W. (2026). Metagenomics reveals an interaction among rhizosphere microbial community, soil properties and active ingredients in a medicinal crop *Panax notoginseng*. J. Ginseng Res..

[10] Cao K., Sun C., Tao J., Zhang L., Pan X., Zhang Y., Shao C., Guan Y., Wang Q. (2026). Differences in ginseng quality and soil characteristics in different growth modes of *Panax ginseng*. Front. Plant Sci..

[11] Pang F., Gao Y., Zhuang Z., Li C., Zhao Y., Liu Q. (2025). Microecological mechanisms of mountainous forest cultivated ginseng growth vigor and saponin accumulation, and the characterization of bionic microbial fertilizer. Front. Microbiol..

[12] Li Z., Xu H., Liu C., Qiao S., Yu Y., Song J., Jin Y., Liu M., Pang S., Li H. (2025). Lifespan- and cultivation-driven ginsenoside dynamics in *Panax ginseng*: Chemical insights into harvest optimization and pharmacological variation. Phytomedicine.

[13] Wang Y., Han L., Li G., Cheng L., Yang K., Li G., Wang E. (2024). Research on the differences in character identification, chemical compositions and pharmacological effects of mountain-cultivated ginseng and farm-cultivated ginseng. Jilin J. Chin. Med..

[14] Zhu H., Zhang R., Huang Z., Zhou J. (2023). Progress in the conversion of ginsenoside Rb1 into minor ginsenosides using β-glucosidases. Foods.

[15] Murthy H.N., Georgiev M.I., Kim Y.S., Jeong C.S., Kim S.J., Park S.Y., Paek K.Y. (2014). Ginsenosides: Prospective for sustainable biotechnological production. Appl. Microbiol. Biotechnol..

[16] Tricco A.C., Lillie E., Zarin W., O’Brien K.K., Colquhoun H., Levac D., Moher D., Peters M.D.J., Horsley T., Weeks L. (2018). PRISMA extension for scoping reviews (PRISMA-ScR): Checklist and explanation. Ann. Intern. Med..

[17] Lee G., Nguyen T.T.H., Lim T.Y., Lim J., Park B., Lee S., Mok I.-K., Pal K., Lim S., Kim D. (2020). Fermented wild ginseng by *Rhizopus oligosporus* improved l-carnitine and ginsenoside contents. Molecules.

[18] Liu Z., Moore R., Gao Y., Chen P., Yu L., Zhang M., Sun J. (2022). Comparison of phytochemical profiles of wild and cultivated American ginseng using metabolomics by ultra-high performance liquid chromatography-high-resolution mass spectrometry. Molecules.

[19] Jeong H.S., Lim C.S., Cha B.C., Choi S.H., Kwon K.R. (2010). Component analysis of cultivated ginseng, cultivated wild ginseng, and wild ginseng and the change of ginsenoside components in the process of red ginseng. J. Korean Pharmacopunct. Inst..

[20] Zhang X., Ran X., Xi Y., Dou D. (2025). The distinction of chemical profiles of mountainous forest cultivated ginseng and garden ginseng based on calcium oxalate crystals, organic acids, and ginsenosides. Foods.

[21] Xu X.F., Cheng X.L., Lin Q.H., Li S.S., Jia Z., Han T., Lin R.C., Wang D., Wei F., Li X.R. (2016). Identification of mountain-cultivated ginseng and cultivated ginseng using UPLC/oa-TOF MSE with a multivariate statistical sample-profiling strategy. J. Ginseng Res..

[22] Fang X., Wang M., Zhou X., Wang H., Wang H., Xiao H. (2022). Effects of growth years on ginsenoside biosynthesis of wild ginseng and cultivated ginseng. BMC Genom..

[23] Kim J., Yun Y., Huh J., Um Y., Shim D. (2023). Comparative transcriptome analysis on wild-simulated ginseng of different age revealed possible mechanism of ginsenoside accumulation. Plant Physiol. Biochem..

[24] Chen J., Yuan Y., Ran X., Guo N., Dou D. (2018). Metabolomics analysis based on a UPLC-Q-TOF-MS metabolomics approach to compare Lin-Xia-Shan-Shen and garden ginseng. RSC Adv..

[25] Yan X.R., Liu Y.X., Xiong B.J., Zhang A., Huang Y.G., He S., He X.H., Shi R. (2023). Comparative study on terpenoids of *Panax ginseng* under different cultivation modes. J. Yunnan Agric. Univ. (Nat. Sci.).

[26] Lee H.Y., Jung J.G., Kim S.C., Cho D.Y., Kim M.J., Lee A.R., Son K.-H., Lee J.H., Lee D.-H., Cho K.M. (2021). Comprehensive comparison of nutritional constituents and antioxidant activity of cultivated ginseng, mountain-cultivated ginseng, and whole plant parts of mountain-cultivated ginseng. J. Appl. Biol. Chem..

[27] Liu S., Wu D., Ma W., Wang T., Yan B., Ge Y., Xiong F., Wang H., Kang C. (2026). Metabolomics and microbiomics reveal the cultivation-dependent divergence in ginsenoside biosynthesis and rhizosphere ecology of *Panax ginseng*. Metabolites.

[28] Li S., Wang P., Yang W., Zhao C., Zhang L., Zhang J., Qin Y., Xu H., Huang L. (2021). Characterization of the components and pharmacological effects of mountain-cultivated ginseng and garden ginseng based on the integrative pharmacology strategy. Front. Pharmacol..

[29] He S.Y., Li W., Zheng Y.N., Wang Q.J. (2010). Comparative analysis of adenosine and ginsenoside contents in forest ginseng and garden ginseng. Chin. J. Pharm. Anal..

[30] Xu L., Xu J., Shi G., Xiao S., Dai R., Wu S., Sun B., Zhang X., Zhao Y. (2020). Optimization of flash extraction, separation of ginsenosides, identification by HPLC-FT-ICR-MS and determination of rare ginsenosides in mountain cultivated ginseng. RSC Adv..

[31] Suh H., Seo S.M., Lee D., Woo S.Y. (2010). Ginsenosides contents of forest cultivated ginseng (*Panax ginseng*) in Korea. For. Sci. Technol..

[32] Zhu H., Lin H., Tan J., Wang C., Wang H., Wu F., Dong Q., Liu Y., Li P., Liu J. (2018). UPLC-QTOF/MS-Based nontargeted metabolomic analysis of Mountain- and garden-cultivated ginseng of different ages in Northeast China. Molecules.

[33] Wang H.P., Zhang Y.B., Yang X.W., Zhao D.Q., Wang Y.P. (2016). Rapid characterization of ginsenosides in the roots and rhizomes of *Panax ginseng* by UPLC-DAD-QTOF-MS/MS and simultaneous determination of 19 ginsenosides by HPLC-ESI-MS. J. Ginseng Res..

[34] Li L., Wang Y., Xiu Y., Liu S. (2018). Chemical differentiation and quantitative analysis of different types of *Panax* genus stem-leaf based on a UPLC-Q-Exactive Orbitrap/MS combined with multivariate statistical analysis approach. J. Anal. Methods Chem..

[35] Zeng C.-Y., Dai Q.-K., Wu H.-Z., Li A., Chen Y.-L., He Q.-H., Tang D., Ma H.-Y. (2026). Comparative analysis of chemical compositional profiles and immunomodulatory activity of ginseng in different habitats. J. Nat. Med..

[36] Zhu L., Xu L., Dou D., Huang L. (2021). The distinct of chemical profiles of mountainous forest cultivated ginseng and garden ginseng based on ginsenosides and oligosaccharides. J. Food Compos. Anal..

[37] Liang J., Chen L., Guo Y., Zhang M., Gao Y. (2019). Simultaneous determination and analysis of major ginsenosides in wild American ginseng grown in Tennessee. Chem. Biodivers..

[38] Yun Y.B., Park M., Lee Y., Um Y. (2025). Investigation of growth and ginsenoside content of wild-simulated ginseng cultivated in different vegetation environments for establishing a plant growth model. Plants.

[39] Kim J.S. (2022). Optimization of accelerated solvent extraction of ginsenosides from cultivated wild ginseng using response surface methodology. Prev. Nutr. Food Sci..

[40] Ahn I.O., Lee S.S., Lee J.H., Lee M.J., Cho B.K. (2008). Comparison of ginsenoside contents and pattern similarity in five cultivars of *Panax ginseng*. J. Ginseng Res..

[41] Rabas A., Colo A., Kaberi K., Ivanov D.A., Bernards M.A. (2025). The properties of ginsenosides in ginseng garden soil: Accumulation, persistence and behaviour. Plant Soil.

[42] Guo N., Yang Y., Yang X., Guan Y., Yang J., Quan J., Yan H., Hou W., Zhang G. (2021). Growth age of mountain cultivated ginseng affects its chemical composition. Ind. Crops Prod..

[43] Chang X., Zhang J., Li D., Zhou D., Zhang Y., Wang J., Hu B., Ju A., Ye Z. (2017). Nontargeted metabolomics approach for the differentiation of cultivation ages of mountain cultivated ginseng leaves using UHPLC/QTOF-MS. J. Pharm. Biomed. Anal..

[44] Chen G., Zhang H., Jiang J., Chen S., Zhang H., Zhang G., Zheng C., Xu H. (2023). Metabolomics approach to growth-age discrimination in mountain-cultivated ginseng (*Panax ginseng* C. A. Meyer) using ultra-high-performance liquid chromatography coupled with quadrupole-time-of-flight mass spectrometry. J. Sep. Sci..

[45] Wang F., Xiao S. (2025). Increment of ginsenoside accumulation in ginseng rhizome joints and the prediction of the growth year. Phytochem. Anal..

[46] Jeong B.G., Jung G.R., Kim M.S., Moon H.G., Park S.J., Chun J. (2019). Ginsenoside contents and antioxidant activities of cultivated mountain ginseng (*Panax ginseng* C.A. Meyer) with different ages. Korean J. Food Preserv..

[47] Yun Y.B., Huh J.H., Jeong D.H., Kim J., Um Y. (2022). Correlation analysis between growth characteristics and ginsenoside contents of 4-year-old wild-simulated ginseng (*Panax ginseng* C.A. Meyer) with different cultivation sites. J. Appl. Biol. Chem..

[48] Piao X.M., Huo Y., Kang J.P., Mathiyalagan R., Zhang H., Yang D.U., Kim M., Yang D.C., Kang S.C., Wang Y.P. (2020). Diversity of ginsenoside profiles produced by various processing technologies. Molecules.

[49] Lee S.M., Bae B.-S., Park H.-W., Ahn N.-G., Cho B.-G., Cho Y.-L., Kwak Y.-S. (2015). Characterization of Korean red ginseng (*Panax ginseng* Meyer): History, preparation method, and chemical composition. J. Ginseng Res..

[50] Zhang C., Yu H., Bao Y., An L., Jin F. (2001). Purification and characterization of ginsenoside-β-glucosidase from ginseng. Chem. Pharm. Bull..

[51] Fu Y., Yin Z.-H., Yin C.-Y. (2017). Biotransformation of ginsenoside Rb1 to ginsenoside Rg3 by endophytic bacterium *Burkholderia* sp. GE 17-7 isolated from *Panax ginseng*. J. Appl. Microbiol..

[52] Xu X.F., Xu S.Y., Zhang Y., Zhang H., Liu M.N., Liu H., Gao Y., Xue X., Xiong H., Lin R.-C. (2017). Chemical comparison of two drying methods of mountain cultivated ginseng by UPLC-QTOF-MS/MS and multivariate statistical analysis. Molecules.

[53] Fan W., Fan L., Wang Z., Mei Y., Liu L., Li L., Yang L., Wang Z. (2024). Rare ginsenosides: A unique perspective of ginseng research. J. Adv. Res..

[54] Zhang L.-L., Gao W.-Y., Song Z.-H., Cai N., Zhou S.-P. (2012). Research on ginsenosides composition in wild ginseng leaves in different growing years. Zhongguo Zhong Yao Za Zhi.

[55] Zhu L., Xu L., Huang Y., Xie C., Dou D., Xu J. (2022). Correlations between ecological factors and the chemical compositions of mountainous forest cultivated ginseng. J. Food Compos. Anal..

[56] Zhu L., Xu J., Dou P., Dou D., Huang L. (2021). The rhizosphere soil factors on the quality of wild-cultivated herb and its origin traceability as well as distinguishing from garden-cultivated herb: Mountainous forest cultivated ginseng for example. Ind. Crops Prod..

[57] Yun Y.B., Huh J.H., Um Y. (2022). Correlations among soil properties, growth characteristics, and ginsenoside contents in wild-simulated ginseng with different ages. Forests.

[58] Yun Y.B., Huh J.H., Um Y.R. (2023). Correlation analysis among soil properties, growth characteristics, and ginsenoside contents of wild-simulated ginseng (*Panax ginseng* C.A. Meyer) with different ages. Acta Hortic..

[59] Yun Y.B., Kwon H.Y., Um Y. (2024). Changes in growth characteristics and ginsenoside contents of wild-simulated ginseng with different harvest times in South Korea. Appl. Biol. Chem..

[60] Yun Y.B., Lee S.H., Park M., Yang S.O., Kim Y.H., Choi W.S., Um Y. (2026). Differences in metabolites and ginsenoside contents of wild-simulated ginseng according to harvest time. Sci. Rep..

[61] Yang B.W., Lee J.B., Lee J.M., Jo M.S., Byun J.K., Kim H.C., Ko S.K. (2019). The comparison of seasonal ginsenoside composition contents in Korean wild simulated ginseng (*Panax ginseng*) which were cultivated in different areas and various ages. Nat. Prod. Sci..

[62] Zhang H., Abid S., Ahn J.C., Mathiyalagan R., Kim Y.J., Yang D.C., Wang Y. (2020). Characteristics of *Panax ginseng* cultivars in Korea and China. Molecules.

[63] Lee K.J., Sebastin R., Kim S.-H., Yoo E., Lee S., Cho G.-T., Kang M., Hyun D.Y. (2020). Genetic composition of Korean *ginseng* germplasm by collection area and resource type. Agronomy.

[64] Gil J., Um Y., Byun J.K., Chung J.W., Lee Y., Chung C.M. (2017). Genetic diversity analysis of wood-cultivated ginseng using simple sequence repeat markers. Korean J. Med. Crop Sci..

[65] Lim W., Mudge K.W., Vermeylen F. (2005). Effects of population, age, and cultivation methods on ginsenoside content of wild American ginseng (*Panax quinquefolium*). J. Agric. Food Chem..

[66] In J.G., Kim M.K., Lee O.R., Kim Y.J., Lee B.S., Kim S.Y., Kwon W.S., Yang D.C. (2010). Molecular identification of Korean mountain ginseng using an amplification refractory mutation system (ARMS). J. Ginseng Res..

[67] Lim W., Mudge K., Weston L. (2007). Utilization of RAPD markers to assess genetic diversity of wild populations of North American ginseng (*Panax quinquefolium*). Planta Med..

[68] Wang H., Li G., Kwon W.-S., Yang D.-C. (2016). Development of EST intron-targeting SNP markers for *Panax ginseng* and their application to cultivar authentication. Int. J. Mol. Sci..

[69] Jo I.H., Kim Y.C., Kim D.H., Kim K.H., Hyun T.K., Ryu H., Bang K.H. (2017). Applications of molecular markers in the discrimination of *Panax* species and Korean ginseng cultivars (*Panax ginseng*). J. Ginseng Res..

[70] Kim J., Yoo J.-M., Kim J.S., Kim S.-G., Park J.E., Seok Y.M., Son J.-H., Kim H.J. (2020). Anticancer effect of mountain ginseng on human breast cancer: Comparison with farm-cultivated ginseng. Evid.-Based Complement. Altern. Med..

[71] Kim J.S., Yoo J.M., Park J.E., Kim J., Kim S.G., Seok Y.M., Son J.H., Kim H.J. (2021). Anti-angiogenic effect of mountain ginseng in vitro and in vivo: Comparison with farm-cultivated ginseng. Mol. Med. Rep..

[72] Guo M., Shao S., Wang D., Zhao D., Wang M. (2021). Recent progress in polysaccharides from *Panax ginseng* C. A. Meyer. Food Funct..

[73] Chung I.-M., Lim J.-J., Ahn M.-S., Jeong H.-N., An T.-J., Kim S.-H. (2016). Comparative phenolic compound profiles and antioxidative activity of the fruit, leaves, and roots of Korean ginseng (*Panax ginseng* Meyer) according to cultivation years. J. Ginseng Res..

[74] Ok S., Kang J.S., Kim K.M. (2016). Simultaneous analysis method for polar and non-polar ginsenosides in cultivated wild ginseng by reversed-phase HPLC-CAD. J. Life Sci..

[75] Chen Q., Wang J., Gao Y., Gao X., Yan P. (2023). Optimization of fermentation conditions and product identification of a saponin-producing endophytic fungus. Microorganisms.

[76] Razgonova M.P., Kalenik T.K., Veselov V.V., Zakharenko A.S., Taghizadehghalehjoughi A., Vuță V., Bărbuceanu F., Izotov B.N., Stratidakis A.K., Tsatsakis A.M. (2019). Supercritical green technologies for obtaining ginsenosides from far-eastern wild ginseng *Panax ginseng* Meyer using SFE for applying in drug, food and cosmetic industries. Farmacia.

[77] Guan Y., Fan J., Sun C., Yang Y., Quan J., Zhang G., Guo N. (2022). Differences in the chemical composition of *Panax ginseng* roots infected with red rust. J. Ethnopharmacol..

[78] Kim C., Choo G.C., Cho H.S., Lim J.T. (2015). Soil properties of cultivation sites for mountain-cultivated ginseng at local level. J. Ginseng Res..

[79] Kim K., Yun Y.B., Park M., Um Y. (2025). Correlation between soil properties and growth of wild-simulated ginseng for site suitability assessment. Korean J. Soil Sci. Fert..

[80] Gao Y., Chen G., Cheng Y., Zhang Y., Wang Y., Liang X., García-Caparros P., Guo Z. (2026). Site and soil drivers of growth and ginsenoside accumulation in forest-grown ginseng. J. Agric. Food Res..

[81] Yong S.H., Seo Y.R., Kim H.G., Park D., Seol Y., Choi E., Hong J.H., Choi M.S. (2020). Growth characteristics and saponin content of mountain-cultivated ginseng (*Panax ginseng* C. A. Meyer) according to seed-sowing method suitable for cultivation under forest. For. Sci. Technol..

[82] Hwang K.H., Kim H.G., Jang K., Kim Y.J. (2024). Novel cultivation of six-year-old Korean ginseng (*Panax ginseng*) in pot: From non-agrochemical management to increased ginsenoside. J. Ginseng Res..

[83] Fang X., Wang H., Zhao L., Wang M., Sun M. (2022). Diversity and structure of the rhizosphere microbial communities of wild and cultivated ginseng. BMC Microbiol..

[84] Bian X., Xiao S., Zhao Y., Xu Y., Yang H., Zhang L. (2020). Comparative analysis of rhizosphere soil physiochemical characteristics and microbial communities between rusty and healthy ginseng root. Sci. Rep..

[85] Xiao C., Yang L., Zhang L., Liu C., Han M. (2016). Effects of cultivation ages and modes on microbial diversity in the rhizosphere soil of *Panax ginseng*. J. Ginseng Res..

[86] He C., Wang R., Ding W., Li Y. (2022). Effects of cultivation soils and ages on microbiome in the rhizosphere soil of *Panax ginseng*. Appl. Soil Ecol..

[87] Bao Y., Qi B., Huang W., Liu B., Li Y. (2020). The fungal community in non-rhizosphere soil of *Panax ginseng* are driven by different cultivation modes and increased cultivation periods. PeerJ.

[88] Huang J., Wu Y., Gao Q., Li X., Zeng Y., Guo Y., Zhang H., Qin Z. (2024). Metagenomic exploration of the rhizosphere soil microbial community and their significance in facilitating the development of wild-simulated ginseng. Appl. Environ. Microbiol..

[89] Fang J., Xu Z.F., Zhang T., Chen C.B., Liu C.S., Liu R., Chen Y.-Q. (2024). Effects of soil microbial ecology on ginsenoside accumulation in *Panax ginseng* across different cultivation years. Ind. Crops Prod..

[90] Xu Z., Chen Y., Sui J., Wang Y., Zhang Q., Zhang T., Chen C. (2025). Effects of rhizosphere soil microecology on the content of ginsenosides in *Panax ginseng*. Rhizosphere.

[91] Khan Chowdhury M.E., Jeon J., Ok Rim S., Park Y.-H., Kyu Lee S., Bae H. (2017). Composition, diversity and bioactivity of culturable bacterial endophytes in mountain-cultivated ginseng in Korea. Sci. Rep..

[92] Park Y.H., Kim Y., Mishra R.C., Bae H. (2017). Fungal endophytes inhabiting mountain-cultivated ginseng (*Panax ginseng* Meyer): Diversity and biocontrol activity against ginseng pathogens. Sci. Rep..

[93] Eo J.K., Choi M.S., Eom A.H. (2014). Diversity of endophytic fungi isolated from Korean ginseng leaves. Mycobiology.

[94] Chowdhury M.E.K., Bae H. (2018). Bacterial endophytes isolated from mountain-cultivated ginseng (*Panax ginseng* Mayer) have biocontrol potential against ginseng pathogens. Biol. Control.

[95] Kim H., Mohanta T.K., Park Y.H., Park S.C., Shanmugam G., Park J.S., Jeon J., Bae H. (2020). Complete genome sequence of the mountain-cultivated ginseng endophyte *Burkholderia stabilis* and its antimicrobial compounds against ginseng root rot disease. Biol. Control.

[96] Park Y.-H., Chandra Mishra R., Yoon S., Kim H., Park C., Seo S.-T., Bae H. (2019). Endophytic *Trichoderma citrinoviride* isolated from mountain-cultivated ginseng (*Panax ginseng*) has great potential as a biocontrol agent against ginseng pathogens. J. Ginseng Res..

[97] Shanmugam G., Dubey A., Ponpandian L.N., Rim S.O., Seo S.T., Bae H., Jeon J. (2018). Genomic insights into nematicidal activity of a bacterial endophyte, *Raoultella ornithinolytica* MG against pine wilt nematode. Plant Pathol. J..

[98] Song X., Wu H., Yin Z., Lian M., Yin C. (2017). Endophytic bacteria isolated from *Panax ginseng* improves ginsenoside accumulation in adventitious ginseng root culture. Molecules.

[99] Lee S.H., Yun Y.B., Kim D.S., Park M., Um Y., Kang J.W. (2025). Isolation of plant growth-promoting rhizobacteria from wild-simulated ginseng and evaluation of soil health following its application in the field. Front. Microbiol..

[100] Lee K.H., Lee K.J., Park H. (2006). The rate and morphology of mycorrhizal infection in the wild ginseng (*Panax ginseng* C.A. Meyer) collected from various locations in Korea. J. Ginseng Res..

[101] Xin J., Lan S., Zhu C., Zhou T. (2026). Ginsenoside-mediated autotoxicity in *Panax ginseng*: Molecular mechanisms, rhizosphere interactions, and management strategies. Plant Soil.

[102] Goodwin P.H., Zaro G. (2024). Appearance and persistence of activity in soil extracts increasing root rot of American ginseng (*Panax quinquefolius*) by *Ilyonectria mors-panacis*. Soil Syst..

[103] Colo A., Bernards M.A. (2025). Influence of ginsenosides on the pathogenicity of *Ilyonectria* spp. J. Phytopathol..

[104] Wang R., Zhou T., Wang Y., Dong J., Bai Y., Huang X., Chen C. (2024). Exploring the allelopathic autotoxicity mechanism of ginsenosides accumulation under ginseng decomposition based on integrated analysis of transcriptomics and metabolomics. Front. Bioeng. Biotechnol..

[105] Dong L., Xu J., Li Y., Fang H., Niu W., Li X., Zhang Y., Ding W., Chen S. (2018). Manipulation of microbial community in the rhizosphere alleviates the replanting issues in *Panax ginseng*. Soil Biol. Biochem..

[106] Jiao X.L., Zhang X.S., Lu X.H., Qin R., Bi Y.M., Gao W.W. (2019). Effects of maize rotation on the physicochemical properties and microbial communities of American ginseng cultivated soil. Sci. Rep..

[107] Zhang J., Zhou D., Yuan X., Xu Y., Chen C., Zhao L. (2022). Soil microbiome and metabolome analysis reveals beneficial effects of ginseng–celandine rotation on the rhizosphere soil of ginseng-used fields. Rhizosphere.

[108] Li Y., Jones F.G., Zhang B., Cui J., Zhang W. (2023). The effect of short-term fallowing on the microbial communities in forest soil cultivated with ginseng: Preliminary research. PeerJ.

[109] Lee H.Y., Lee J.H., Shin E.C., Cho D.Y., Jung J.G., Kim M.J., Jeong J.B., Kang D., Kang S.S., Cho K.M. (2022). Changes in chemical compositions and antioxidant activities from fresh to fermented red mountain-cultivated ginseng. Molecules.

[110] Kim G.N. (2023). Changes of ginsenoside compositions in cultivated wild ginseng with different steaming temperatures and time. J. East Asian Soc. Diet. Life.

[111] Cha B.C., Yoon H.C., Lee D.H., Park J.S., Kwon K.R. (2010). Component analysis of cultivated ginseng and mountain ginseng to the change of ginsenoside components in the process of heating and fermentation. J. Pharmacopunct..

[112] Lee J.H., Kwon K.R., Cha B.C. (2008). Component analysis of cultivated ginseng, red ginseng, cultivated wild ginseng, and red wild ginseng using HPLC method. J. Korean Inst. Herb. Acupunct..

[113] Guo B., Liang Y., Fu B., Luo J., Zhou X., Ji R., He X. (2024). Integrated analysis of ginsenoside content and biomarker changes in processed ginseng: Implications for anti-cancer mechanisms. Foods.

[114] Mok I.K., Jung H.Y., Kim H., Kim D. (2023). Biotransformation of ginsenosides from Korean wild-simulated ginseng (*Panax ginseng* C. A. Mey.) using the combination of high hydrostatic pressure, enzymatic hydrolysis, and sonication. Food Biosci..

[115] Jeon I.H., Cho G.Y., Han S.I., Yoo S.K., Whang K.S. (2013). Conversion of ginsenoside Rb1 and taxonomical characterization of *Stenotrophomonas* sp. 4KR4 from ginseng rhizosphere soil. Korean J. Microbiol..

[116] Murthy H.N., Dandin V.S., Park S.Y., Paek K.Y. (2018). Quality, safety and efficacy profiling of ginseng adventitious roots produced in vitro. Appl. Microbiol. Biotechnol..

[117] Zhang J.Y., Bae T.W., Boo K.H., Sun H.J., Song I.J., Pham C.H., Ganesan M., Yang D.H., Kang H.G., Ko S.M. (2011). Ginsenoside production and morphological characterization of wild ginseng (*Panax ginseng* Meyer) mutant lines induced by γ-irradiation ((60)Co) of adventitious roots. J. Ginseng Res..

[118] Langhansová L., Marsik P., Vanek T. (2005). Production of saponins from *Panax ginseng* suspension and adventitious root cultures. Biol. Plant..

[119] Wang J., Liu H., Gao W.-Y., Zhang L. (2013). Comparison of ginsenoside composition in native roots and cultured callus cells of *Panax quinquefolium* L. Acta Physiol. Plant..

[120] Zhao N., Cheng M., Lv W., Wu Y., Liu D., Zhang X. (2020). Peptides as potential biomarkers for authentication of mountain-cultivated ginseng and cultivated ginseng of different ages using UPLC-HRMS. J. Agric. Food Chem..

[121] Tajudeen H., Mun J., Ha S., Hosseindoust A., Lee S., Kim J. (2023). Effect of wild ginseng on the laying performance, egg quality, cytokine expression, ginsenoside concentration, and microflora quantity of laying hens. J. Anim. Sci. Technol..

[122] Yang W., Zhou J., Harindintwali J.D., Yu X. (2021). Production of minor ginsenosides by combining *Stereum hirsutum* and cellulase. PLoS ONE.

