# Supplementary material for: Ginsenoside Profiles of Wild-Simulated and Forest-Grown Panax ginseng: A Scoping Review of Quantitative Evidence and Confounding Factors
Source: Plants (Basel). 2026 Nov 6;15(21):3472. doi: 10.3390/plants15213472 (PMC13766043; doi:10.3390/plants15213472)
Supplement: Supplementary file 1 [file plants-15-03472-s001.zip › Supplementary Materials_revised.pdf]

Supplementary Materials

Ginsenoside Profiles of Wild-Simulated and Forest-Grown *Panax ginseng*: A Scoping Review of Quantitative Evidence and Confounding Factors

Min-Kyu Park, Jong-Du Lee, Gun-Myung Lee, Yeong-Jun Park, Xiaofei Dang, Yuxin He, *Hyuk*-Joon *Kwon and Jae-Ho Shin*

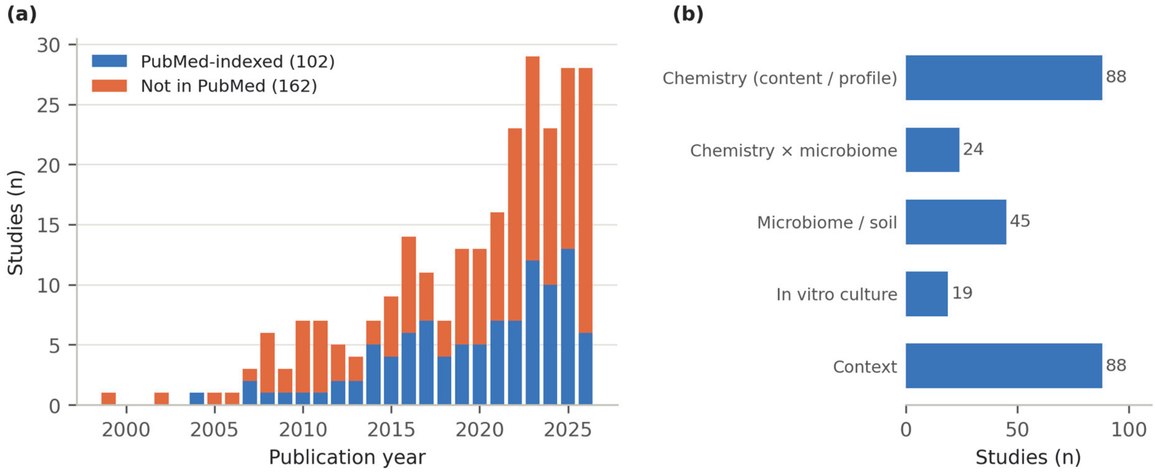

Figure S1. Composition of the scoping corpus (264 unique studies; the 270 retained records include six pairs merged at the charting stage because they shared a DOI or a near-identical title). (a) Publication year by retrieval route; 61% of the 264 studies were not retrieved by the PubMed queries (this reflects the reach of the queries, not MEDLINE indexing status). (b) Topic categories used for charting; each study is assigned to one category here (chemistry × microbiome denotes studies charted in both).

Table S2. Complete search strategies for the four search arms. No date or language limits were applied. The PubMed field tag [tiab] restricts terms to title and abstract.

Arm	Source	Date run	Search string / procedure	Records
1	PubMed (E-utilities esearch)	14 August 2026	("wild ginseng"[tiab] OR "mountain-cultivated ginseng"[tiab] OR "mountain cultivated ginseng"[tiab] OR "forest-cultivated ginseng"[tiab] OR "wild-simulated ginseng"[tiab] OR "mountainous forest cultivated ginseng"[tiab] OR "mountainous forest-cultivated ginseng"[tiab] OR "forest ginseng"[tiab] OR "under-forest ginseng"[tiab] OR "understory-cultivated ginseng"[tiab] OR "forest-grown ginseng"[tiab] OR "wild-grown ginseng"[tiab] OR "mountain ginseng"[tiab] OR "wild mountain ginseng"[tiab]) AND (ginsenoside[tiab] OR ginsenosides[tiab] OR saponin*[tiab])	91
2	PubMed (E-utilities esearch)	14 August 2026	("wild ginseng"[tiab] OR "mountain-cultivated ginseng"[tiab] OR "mountain cultivated ginseng"[tiab] OR "forest-cultivated ginseng"[tiab] OR "wild-simulated ginseng"[tiab] OR "mountainous forest cultivated ginseng"[tiab] OR "mountainous forest-cultivated ginseng"[tiab] OR "forest ginseng"[tiab] OR "under-forest ginseng"[tiab] OR "understory-cultivated ginseng"[tiab] OR "forest-grown ginseng"[tiab] OR "wild-grown ginseng"[tiab] OR "mountain ginseng"[tiab] OR "wild mountain ginseng"[tiab]) AND (microbiome[tiab] OR microbiota[tiab] OR	23

			rhizospher*[tiab] OR endophyt*[tiab] OR "soil bacteri*"[tiab] OR "bacterial community"[tiab] OR "fungal community"[tiab] OR "microbial communit*"[tiab] OR PGPR[tiab] OR "plant growth-promoting"[tiab])	
3	Crossref REST API (query.bibliographic)	14 August 2026	One query per phrase (hyphens replaced by spaces; rows = 100 per query): "forest cultivated ginseng"; "forest ginseng"; "forest grown ginseng"; "mountain cultivated ginseng"; "mountain ginseng"; "mountainous forest cultivated ginseng"; "under forest ginseng"; "understory cultivated ginseng"; "wild ginseng"; "wild grown ginseng"; "wild mountain ginseng"; "wild simulated ginseng". Records were retained only if the normalized title contained one of the phrases. Three additional microbiome queries: "wild simulated ginseng ginsenoside rhizosphere soil microbial", "mountain cultivated ginseng ginsenoside rhizosphere soil microbial", and "forest cultivated ginseng ginsenoside rhizosphere soil microbial", retaining titles that contained "ginseng" and one of: rhizosphere, microbiome, microbial, soil bacteri-, endophyt-, bacterial community, fungal community, microbiota.	220
4	Hand-searching	Up to 14 August 2026	Korean-language journals indexed in the Korea Citation Index (KCI) and known papers in journals not indexed in MEDLINE, identified from reference lists and journal tables of contents; each record entered manually with its bibliographic data.	18
—	De-duplication	14 August 2026	Normalized DOI, then normalized title (case, punctuation and markup removed).	352 → 300
