# Supplementary material for: Ginsenoside Profiles of Wild-Simulated and Forest-Grown Panax ginseng: A Scoping Review of Quantitative Evidence and Confounding Factors
Source: Plants (Basel). 2026 Nov 6;15(21):3472. doi: 10.3390/plants15213472 (PMC13766043; doi:10.3390/plants15213472)
Supplement: Supplementary file 1 [file plants-15-03472-s001.zip › Table S3_PRISMA-ScR checklist.pdf]

Table S3. Completed PRISMA-ScR checklist

Manuscript: Ginsenoside Profiles of Wild-Simulated and Forest-Grown *Panax ginseng*: A Scoping Review of Quantitative Evidence and Confounding Factors

Checklist items follow Tricco et al., Ann. Intern. Med. 2018, 169, 467–473 (PRISMA-ScR). Locations refer to sections, tables and figures of the revised *manuscript.*

Section / topic	#	PRISMA-ScR checklist item	Reported in
**TITLE**			
Title	1	Identify the report as a scoping review.	Title
**ABSTRACT**			
Structured summary	2	Background, objectives, eligibility criteria, sources of evidence, charting methods, results and conclusions that relate to the review questions and objectives.	Abstract
**INTRODUCTION**			
Rationale	3	Rationale for the review in the context of what is already known.	Section 1
Objectives	4	Explicit statement of the questions and objectives being addressed.	Section 1 (four review questions)
**METHODS**			
Protocol and registration	5	Indicate whether a review protocol exists and where it can be accessed; if available, provide registration information.	Section 2.1 (the review protocol was not registered in advance); Section 2.3
Eligibility criteria	6	Characteristics of the sources of evidence used as eligibility criteria, with rationale.	Section 2.1 (eligibility criteria and exclusion criteria E1–E5); Section 2.2 (criteria for quantitative charting)
Information sources	7	Describe all information sources and the date the most recent search was executed.	Section 2.1 (PubMed, Crossref, hand-searching; all searches run on 14 August 2026); Table S2
Search	8	Present the full electronic search strategy for at least one database.	Table S2 (complete search strings for all four arms)
Selection of sources of evidence	9	State the process for selecting sources of evidence (screening and eligibility).	Section 2.1 (rule-based screening, each decision verified by one reviewer); Section 2.2 (two-step full-text eligibility); Figure 1
Data charting process	10	Describe the methods of charting data from the included sources.	Section 2.2 (values transcribed by one author and re-checked against the source by a second author)
Data items	11	List and define all variables for which data were sought.	Section 2.2; Table S1 (19-field schema with data dictionary)
Critical appraisal of individual sources (optional)	12	If done, provide a rationale and describe the methods used.	Formal appraisal not performed; internal inconsistencies of published tables recorded (Section 2.2; Appendix

			A)
Synthesis of results	13	Describe the methods of handling and summarizing the data that were charted.	Section 2.2 (study as unit of synthesis; direction tallies; no meta-analysis)
**RESULTS**			
Selection of sources of evidence	14	Numbers of sources screened, assessed and included, with reasons for exclusions, ideally using a flow diagram.	Sections 2.1 and 2.2; Figure 1
Characteristics of sources of evidence	15	For each source of evidence, present characteristics for which data were charted.	Tables 2 and 3; Figure S1; Table S1
Critical appraisal within sources of evidence (optional)	16	If done, present data on critical appraisal of included sources.	Appendix A
Results of individual sources of evidence	17	For each included source, present the relevant data that were charted.	Tables 2–4; Figures 2 and 3; Table S1
Synthesis of results	18	Summarize and/or present the charting results as they relate to the review questions and objectives.	Sections 5–10 (including Section 5.6); Figures 2–4
**DISCUSSION**			
Summary of evidence	19	Summarize the main results, link them to the review questions, and consider relevance to key groups.	Sections 5.6, 11 and 13
Limitations	20	Discuss the limitations of the scoping review process.	Section 2.3 (limitations of the search and charting); Section 5.1 (weight of evidence); Section 6
Conclusions	21	Provide a general interpretation of the results and potential implications and/or next steps.	Sections 12 and 13
**FUNDING**			
Funding	22	Describe sources of funding and the role of funders.	Funding statement (back matter)
